# MTUS2-AS1 suppression promotes DDX5 protein degradation to enhance the sensitivity of PARP inhibitors in BRCA-wild triple negative breast cancer

**DOI:** 10.7150/ijbs.133410

**Published:** 2026-07-20

**Authors:** Tao Xu, Junjie Nie, Bei Pan, Qiwei Hong, Yujing Fan, Lei Dong, Jian Qin, Huiling Sun, Mu Xu, Yuqin Pan, Shukui Wang

**Affiliations:** 1 General Clinical Research Center, Nanjing First Hospital, Nanjing Medical University, Nanjing 210006, Jiangsu, China.; 2 Department of Laboratory Medicine, Nanjing First Hospital, Nanjing Medical University, Nanjing 210006, Jiangsu, China.; 3 Department of Clinical Pharmacy, School of Basic Medicine and Clinical Pharmacy, China Pharmaceutical University, Nanjing 210006, Jiangsu, China.; 4 Department of Laboratory Medicine, Ganyu District People's Hospital, Kangda College of Nanjing Medical University, Lianyungang 222100, Jiangsu, China.; 5 Jiangsu Cancer Personalized Medicine Collaborative Innovation Center, Nanjing 211112, Jiangsu, China.; 6 Nanjing Medical Key Laboratory of Laboratory Diagnostics, Nanjing 211112, Jiangsu, China.

**Keywords:** triple-negative breast cancer, long non-coding RNA, PARP inhibitor, DNA damage, DDX5

## Abstract

Triple-negative breast cancer (TNBC) primarily relies on traditional adjuvant chemotherapy and radiotherapy, which have significant side effects and are prone to drug resistance. Poly ADP-ribose polymerase inhibitor (PARPi) has been approved for TNBC patients with BRCA mutation, but some BRCA wild-type patients with homologous recombination deficiencies are also sensitive to PARPi. Therefore, it is important to identify potential molecules that influence PARPi sensitivity in BRCA wild-type TNBC and to explore their specific mechanisms. Through CRISPR-cas9 loss-of-function screening, the lncRNA MTUS2-AS1 was identified to be significantly correlated with PARPi sensitivity in BRCA wild-type TNBC cells. *In vitro* and *in vivo* experiments were performed to investigate its function and underlying mechanism. We found that MTUS2-AS1 knockdown suppressed DNA damage repair and enhanced the anticancer effects of PARPi in BRCA-wild TNBC cells. Mechanistically, MTUS2-AS1 upregulates DDX5 protein expression by maintaining its stability, thereby promoting R-loop resolution, and suppressing DNA damage. Knocking down MTUS2-AS1 accelerated DDX5 protein degradation, reduced DDX5 protein expression, inhibitd R-loop resolution, promoted DNA damage, and ultimately enhanced the PARPi sensitivity in TNBC cells. Our study provided new insights into exploring the key molecules and mechanisms influencing the sensitivity of PARPi treatment and had great significance for screening benefit population and expanding the indications of PARPi treatment.

## Introduction

Breast cancer (BC) remains the most commonly diagnosed malignancy and the second leading cause of cancer-related deaths among women worldwide^1^. Triple negative breast cancer (TNBC), the most malignant subtype of BC, is characterized by the lack of estrogen receptor (ER), progesterone receptor (PR), and human epidermal growth factor receptor 2 (HER2) expression, accounting for approximately 15-20% of all BC cases^2^. Due to the absence of effective therapeutic targets, TNBC primarily relies on conventional surgery combined with adjuvant chemotherapy and radiotherapy, which have significant side effects and are prone to drug resistance^3^. Given these challenges, the development of novel therapeutic strategies, including molecularly targeted agents, has garnered widespread attention. In recent years, the application of Poly-ADP-ribose polymerase inhibitors (PARPi) and related targeted therapies has brought new hope to TNBC patients. The Food and Drug Administration (FDA) has granted approval to Olaparib and Talazoparib, two typical PARPi, for the treatment of patients with deleterious or suspected deleterious germline BRCA-mutated (gBRCAm), HER2-negative metastatic breast cancer, including TNBC^4^, ^5^. However, the prevalence of germline BRCA1/2 mutations in TNBC is only approximately 10% - 20%^6^, which limits the widespread use of PARPi. Therefore, there is an urgent clinical need to expand the application of PARPi treatment in TNBC patients.

It is reported that PARPi exert their effects by inhibiting PARP enzyme activity and trapping PARP-DNA complexes, thereby preventing the repair of DNA single-strand breaks (SSBs). The accumulation of SSBs damage leads to the collapse of replication forks and the formation of DNA double-strand breaks (DSBs). In cells harboring BRCA mutations, where homologous recombination (HR), an essential pathway for DSBs repair is defective, PARPi exploit synthetic lethality, ultimately inducing tumor cell apoptosis^7^. In addition to loss-of-function mutations in BRCA, deficiencies in other HR-related genes, such as PALB2, CDK12, RAD51C/D, CHEK2, and ATM, result in homologous recombination deficiency (HRD) and confer sensitivity to PARPi^8^. Recent clinical trials have demonstrated that BC patients with wild-type BRCA but PALB2 mutations are likely to benefit from PARPi treatment^9^, ^10^. Furthermore, Ding *et al*. exploited metabolic alterations to induce HRD and sensitize PARPi treatment in TNBC with low HRD scores^11^. Shan *et al*. found that CDK7 inhibition preferentially suppresses the expression of DNA damage signaling genes and impairs HR activity, thereby sensitizing wild-type TNBC cells to PARPi-induced cell death^12^. These emerging studies suggest that PARPi treatment has the potential to be extended to TNBC patients lacking BRCA mutations.

Long non-coding RNAs (lncRNAs) have recently been redefined as non-coding RNAs exceeding 500 nucleotides in length^13^. Well-characterized examples have revealed that lncRNAs play biological roles in regulating drug efficacy in various malignancies, including chemotherapy^14^, immunotherapy^15^, and molecular targeted therapy^16^. Wu *et al*. demonstrated that blockade of the lncRNA PART1 confers resistance to PARPi and promotes cellular senescence in ovarian cancer^17^. Guo *et al*. reported that lncRNA CTD-2256P15.2 encodes a micropeptide that affects sensitivity to PARP/ATR/CDK4/6 inhibitors by modulating DNA damage response^18^. Therefore, identifying potential lncRNA molecules that influence sensitivity to PARPi treatment in BRCA wild-type TNBC and exploring their regulatory mechanisms are of great significance for screening benefit population and expanding the indications of PARPi treatment.

DEAD-box RNA helicase DDX5 (also known as p68), a member of the DEAD-box RNA helicase family, plays multiple biological roles in human cancers^19^. In recent years, the function of DDX5 in genome stability and DNA repair has been the subject of extensive research. Rocchi *et al*. identified a set of DDX5-binding proteins involved in the DNA damage response in castration-resistant prostate cancer (CRPC) cells, and demonstrated that DDX5 inhibition reduces DNA repair efficiency and enhances sensitivity to either irradiation or cisplatin in CRPC DU-145 cells^20^. Yang *et al*. showed that lncRNA SLC26A4-AS1 inhibits the expression of multiple DSBs repair genes and suppresses thyroid cancer metastasis by destabilizing DDX5^21^. Additionally, DDX5 is recognized as a critical regulator for DNA-RNA hybrids (termed R-loop) generated during DNA transcription, replication, and repair. Aberrant accumulation of R-loop causes catastrophic replication conflicts and exacerbates DNA damage^22^. Richard *et al*. confirmed that R-loop accumulation in DDX5-deficient cells causes DSBs, and the unrepaired DSBs further induce cell apoptosis^23^. Carreira *et al*. found that BRCA2-DDX5 interaction stimulates DDX5 activity in R-loop resolution, thereby promoting DNA damage repair^24^. Zheng *et al*. observed that Lnc530-DDX5-TDP-43 complex increases the expression of DDX5 and TDP-43, leading to decreased R-loop formation in mouse embryonic stem cells (mESCs)^25^. Collectively, these studies indicate that DDX5 plays a central role in the accumulation and resolution of R-loop. Moreover, a growing body of research has demonstrated that R-loop formation is associated with the PARPi sensitivity. Nguyen *et al*. found that leukemias with RNA splicing factor gene mutations exhibit R-loop accumulation and are preferentially sensitive to PARPi^26^. Lee *et al*. reported that in PARPi-resistant high-grade serous ovarian cancer, AKT1 interacts with DHX9 to facilitate R-loop resolution, and that AKT inhibitors overcome PARPi resistance by inducing R-loop mediated replication stress through mitigating DHX9 recruitment to R-loop^27^. These findings suggest that R-loop accumulation could enhance PARPi sensitivity.

Herein, we screened for lncRNAs that are specifically and highly expressed in TNBC using the Cancer Genome Atlas (TCGA) database and the Cancer Cell Line Encyclopedia (CCLE) database. We than designed and synthesized a single-guide RNA (sgRNA) library targeting these highly expressed lncRNAs through using a genome-wide CRISPR-cas9 knockout library developed by Wei group^28^, which specifically targets the splicing sites of lncRNAs. After CRISPR-cas9 loss-of-function screening, we identified MTUS2-AS1 as an essential contributor to the insensitivity of BRCA wild-type TNBC cells to PARPi treatment. Mechanistically, MTUS2-AS1 upregulates DDX5 protein expression by maintaining its stability, thereby promoting R-loop resolution, and suppressing DNA damage. Knockdown of MTUS2-AS1 accelerates DDX5 protein degradation, reduces DDX5 protein expression, inhibits R-loop resolution, promotes DNA damage, and ultimately enhances the PARPi sensitivity in TNBC cells. Our study provided new insights into exploring the key molecules and mechanisms influencing PARPi sensitivity, promoting the optimized use of PARPi in TNBC patients.

## Materials and Methods

### Cell lines and cell culture

The normal human mammary epithelial cell line (MCF-10A) and BC cell lines (MCF-7, T47D, MDA-MB-231, BT549, MDA-MB-468, MDA-MB-436, and HCC1937) were all purchased from the American Type Culture Collection (ATCC). Among these, MCF-7 and T47D are non-TNBC cell lines, whereas the remaining BC cell lines (MDA-MB-231, BT549, MDA-MB-468, MDA-MB-436, and HCC1937) are TNBC cell lines. Specifically, MDA-MB-231, BT549, and MDA-MB-468 are BRCA wild-type TNBC cell lines, whereas MDA-MB-436 and HCC1937 are BRCA mutant-type TNBC cell lines. The culture methods for MCF-10A, MCF-7, T47D, MDA-MB-231, BT549, and MDA-MB-468 cell lines were as previously described^29^. HCC1937 cells were maintained in Roswell Park Memorial Institute (RPMI) 1640 medium (KeyGEN, China) supplemented with 10% fetal bovine serum (FBS; ABW, Uruguay). MDA-MB-436 cells were grown in Leibovitz's L-15 medium (KeyGEN, China) supplemented with 20% FBS and 0.01mg/ml insulin (Beyotime, China). All the cell lines were authenticated by Short Tandem Repeat (STR) analysis and tested negative for mycoplasma contamination. The Olaparib-resistant MDA-MB-231 and BT549 cell lines were established through induction in our laboratory, and the resistance index was determined by calculating the half maximal inhibitory concentration (IC50).

### RNA isolation and quantitative polymerase chain reaction (qPCR)

Total RNA was extracted from whole cells using RNA-easy isolation reagent (Vazyme Biotech, China). RNA isolation from nuclear and cytoplasmic fractions was performed with the PARIS^TM^ Protein and RNA isolation System (Invitrogen, USA) following the manufacturer's protocol. Nucleic acid quantification was measured with NanoDrop One spectrophotometer (ThermoFisher, USA). Reverse transcription and qPCR were conducted with ABScript III RT Master Mix for qPCR with gDNA Remover (Abclonal, China) on the Bio-Rad C1000 Touch Thermal Cycler and 2X Universal SYBR Green Fast qPCR Mix (Abclonal, China) on the Applied Biosystems 7500 Sequence Detection System, respectively. GAPDH was used as an internal gene. The sequences of primers used in this study are listed in Supplementary Table S1.

### CRISPR-cas9 loss-of-function screening in MDA-MB-231 cell line

A total of 308 lncRNAs was incorporated into screening in BRCA wild-type MDA-MB-231 cell line. Then, a sgRNA library targeting the 238 out of 308 lncRNAs containing 3706 sgRNAs through employing the genome-wide CRISPR-cas9 knockout library developed by Wei group^28^ was designed and synthesized. All sequences of the sgRNA library were cloned with lentiCRISPR v2 vector (Addgene, #52961) and lentivirus was produced with packaging vectors. MDA-MB-231 cells were infected with lentivirus at a low multiplicity of infection (MOI = 0.3) in the presence of 8 mg/ml polybrene. After 48 h transfection, puromycin (2 μg/ml) was added and maintained for at least a week to select the successfully transfected cells. Then, transfected MDA-MB-231 cells were divided into PARPi Olaparib (6 μM) and DMSO treated arms. After 14 days of treatment, surviving cells in two treated arms were harvested for sgRNA sequencing analysis by the Model-based Analysis of Genome-wide CRISPR-cas9 Knockout (MAGeCK) algorithm^30^.

### RNA chromogenic *in situ* hybridization (CISH)

Fifteen pairs of TNBC tissues and adjacent non-tumor tissues were collected from Nanjing First Hospital. The study was approved by the Institutional Review Board of the Nanjing First Hospital. Then, MTUS2-AS1 expression levels in clinical TNBC samples were measured using a specific digoxigenin-labelled probe. The detailed sequence of this probe was listed in Supplementary Table S1. The CISH assay was performed, and staining was scored based on staining intensity and ratio of positive cells, as described previously^29^.

### Plasmid and short hairpin RNA (shRNA) lentiviral transfection

pLV3-CMV-DDX5-3 × FLAG-CopGFP-Hyg vector was purchased from Miaoling plasmid (Wuhan, China). The third-generation package system was used to generate lentiviruses. Packaged plasmid at a concentration of 1 μg/μl in accordance with the ratio of pMD2.G: pRSV-Rev: pMDLg/pRRE = 1:1:2 was prepared. Then, 1.43 μg of indicated vector and 2.57 μg of packaged plasmid were mixed and transfected into HEK293T cells in six-well plate with ExFect® Transfection Reagent (Vazyme, Nanjing) following the manufacturer's instructions. After 48 h of transfection, the lentiviruses supernatant was collected to infect the target cells. For shRNA lentiviral transfection, the lentiviruses of sh-MTUS2-AS1#1/#2/#3 and negative control were purchased from GenePharma (Shanghai, China). The indicated cells were transfected with corresponding lentiviruses according to the manufacturer's instructions. The detailed shRNA sequnences were listed in Supplementary Table S1.

### Measurement of half maximal inhibitory concentration (IC50)

Approximately 4-8 × 10^3^ cells were seeded in 48-well plates. After 24 h, 100 μl of Olaparib solution was added to each well according to concentration gradients of either 0, 1.5625, 3.125, 6.25, 12.5, 25, 50, 100 μM or 0, 0.1, 0.316, 1, 3.16, 10, 31.6, 100 μM. Each well was supplemented with 300 μl of culture medium. Three replicate wells were setted for each group, and then the plates were placed in cell incubator. The Olaparib-containing medium was replaced every 72 h. After a total of 7 days of growth, cells were stained with crystal violet solution 10% in methanol and photoed. Then, 200 μl of 2% SDS solution was added to each well and incubated on a shaker for 30 min. The absorbance at a wavelength of 570 nm was detected and analyzed.

### Subcellular localization detection

Fluorescence *in situ* hybridization (FISH) assay was carried out using a Fluorescent *In Situ* Hybridization Kit (Ribobio, Guangzhou) according to the manufacturer's protocols. Cell fractionation assay was performed with PARIS Kit (Ambion, Life Technologies) as described previously^29^.

### Cell cycle analysis

Approximately 1 × 10^6^ cells were collected and fixed in 70% ice-cold ethanol at least 18 h. Then, cells were stained with a Cell Cycle Detection Kit (KeyGEN, Jiangsu) and analyzed on a DxFLEX flow cytometer (Beckman Coulter, USA). The data were analyzed with ModFit LT software.

### Immunofluorescence staining

Approximately 2 × 10^4^ cells were seeded on coverslips in 24-well plates and cultured for 24 h. Then, cells were fixed with 4% paraformaldehyde for 15 min and washed three times with PBS at room temperature. Likewise, cells were incubated in 0.5% Triton X-100 solution for 15 min and blocked with 5% BSA buffer for 10 min. Next, the coverslips were mounted onto the glass slides and incubated with primary antibody overnight at 4 ℃. The second day, cells were incubated with a secondary antibody and stained with DAPI. Finally, images were visualized using a Carl Zeiss fluorescence microscope. Phospho-Histone H2AX-S139 Rabbit pAb (AP0099, ABclonal, China), anti-DNA-RNA Hybrid [S9.6] antibody (ENH001, Karafast, USA) and Fibrillarin/U3 RNP Rabbit mAb (A0850, ABclonal, China) were used for immunofluorescence staining.

### Comet assay/Single-cell gel electrophoresis assay

Approximately 1 × 10^5^ cells were added into 1% agarose solution and mixed well. 100 μl of the gel solution containing cells was drop on a comet assay slide (Beyotime, China) and maintained in the dark for 30 min at 4 ℃. The slides were placed in lysis buffer supplemented with 1% Triton X-100 in the dark for 1 h at 4 ℃. Then, the slides were incubated with electrophoresis buffer in the dark for 1 h at 4 ℃. Electrophoresis was performed for 25 min at 25 V. Subsequently, the slides were immersed twice in ddH_2_O for 5 min, once in 70% ethanol for 5 min, dried for 10 min in 37 ℃ oven, and stained with Safe Red (ABclonal, China) in the dark for 30 min. Finally, the slides were washed with ddH_2_O to remove the residual staining solution and dried in 37 ℃ oven until the gel became into a thin film. Images were observed using a Carl Zeiss fluorescence microscope.

### *In vivo* tumor experiments

For xenograft model, approximately 1 × 10^7^ cells were subcutaneously injected into two flanks of each six-week-old female BALB/c nude mouse in the armpit. Tumors were measured with a digital caliper every 5 days once the tumor became palpable. Tumor volume was calculated according to the formula: tumor volume = 0.5 × length × width^2^. The mice were randomly divided into four groups for indicated treatment when tumor volume reached 100 cm^3^. Olaparib treatment was referred to the previous literature^31^, intraperitoneally injected 5 d per week with a dose of 50 mg/kg. After treatment for 3 weeks, the mice were euthanized and tumors were harvested, measured, and analyzed by HE and IHC staining. All animal experiments were approved by the ethics committee of Nanjing First Hospital.

### RNA pull-down assay and RNA immunoprecipitation (RIP)

For RNA pull-down assay, biotin-labeled RNAs were transcribed *in vitro* using Ribo^TM^ RNAmax-T7 Biotin Labeling Transcription Kit (Ribobio, China). The RNA-protein complexes were retrieved with Pierce^TM^ Magnetic RNA-Protein Pull-Down Kit (ThermoFisher, USA) according to the manufacturer's instructions and further detected by mass spectrometry and western blotting. For RIP assay, detailed steps were described previously^29^ using a Magna RIP^TM^ RNA-binding protein immunoprecipitation kit (Millipore, USA).

### Ubiquitination assay

Cells were treated with the proteasome inhibitor MG132 (20 μM, Topscience, China) for 4 h and then lysed with IP buffer. After centrifugation, 50 μl supernatant was gathered as input for the following western blotting. The remaining IP lysate was incubated with indicated antibodies at 4 ℃ with gentle rotation overnight. The next day, 40 µl Protein A/G Plus-Agarose (Santa Cruz, USA) was added to the lysate and incubated at 4 ℃ with gentle rotation for 4-6 h. After five times of wash, proteins were immunoprecipitated and extracted for the following western blotting.

### Protein isolation and western blotting (WB)

Cell proteins were isolated using lysis buffer and quantitated with NanoDrop One spectrophotometer (ThermoFisher, USA). The detailed procedures were carried out according to the protocols as previously reported^32^. GAPDH monoclonal antibody (60004-1-Ig, Proteintech, China), DDX5 Rabbit monoclonal antibody (A11339, ABclonal, China), Phospho-Histone H2AX-S139 Rabbit pAb (AP0099, ABclonal, China), Anti-Ubiquitin Rabbit monoclonal antibody (PTM-7311, PTMBIO, China), HRP-conjugated affinipure goat anti-Mouse IgG(H+L) (SA00001-1, Proteintech, China) and HRP-conjugated affinipure goat anti-Rabbit IgG(H+L) (SA00001-2, Proteintech, China) were used.

### Statistical analysis

All data was analysed using SPSS 22.0 and GraphPad Prism 7.0 softwares. Continuous variables are presented as mean ± standard deviation (SD). Differences between groups were compared using Student's *t*-test or one-way analysis of variance (ANOVA). All tests were two-tailed, and *P *< 0.05 was considered statistically significant. All the experiments were performed at least three times.

## Results

### Identification of lncRNA MTUS2-AS1 involved in sensitivity to PARPi treatment in BRCA wild-type TNBC cells

**Figure 1:**
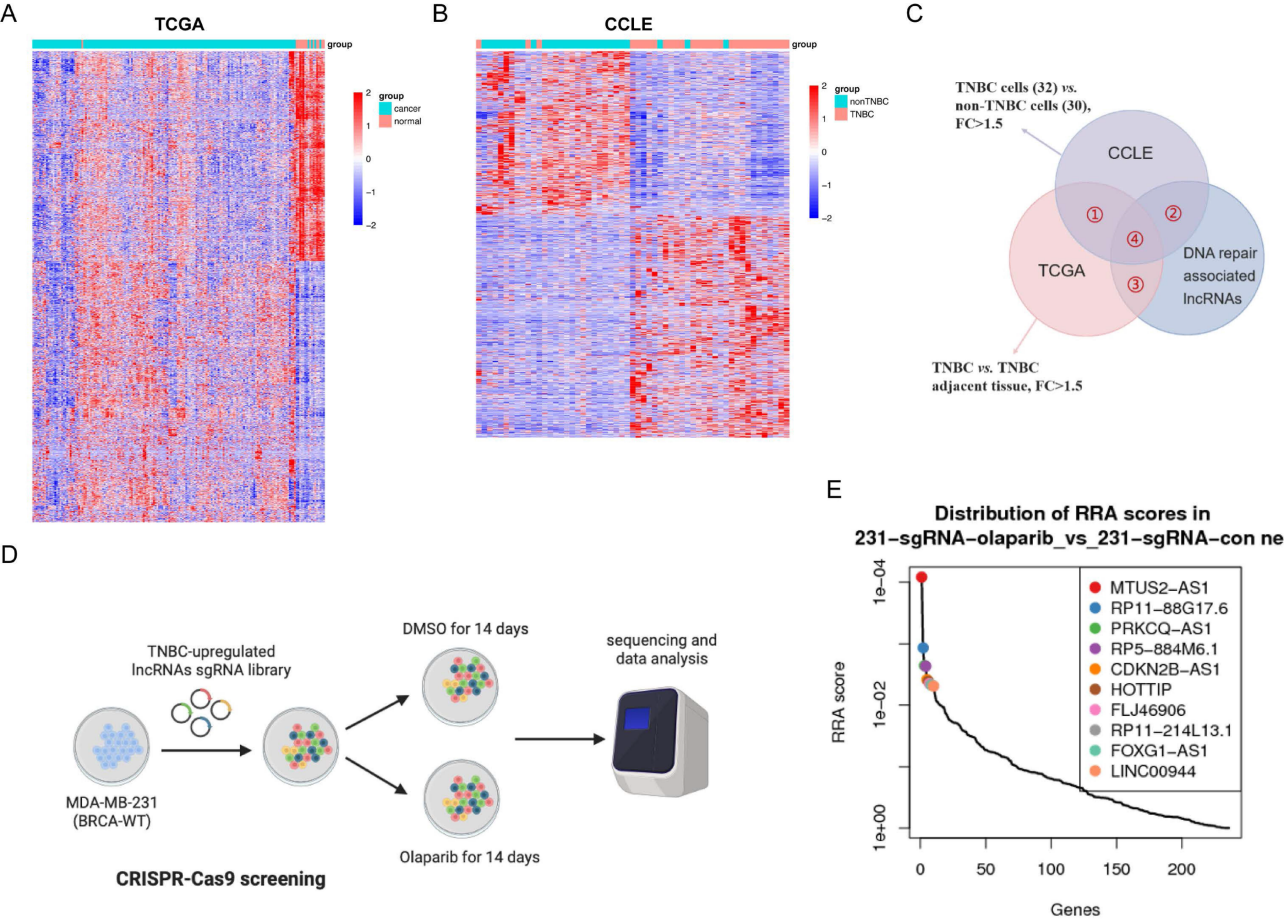
** CRISPR-Cas9 screening identified determinants of PARPi response in BRCA wild-type TNBC cells. A.** Heatmap of differentially expressed lncRNAs between 158 TNBC and 14 adjacent normal tissue samples in TCGA database. **B.** Heatmap of differentially expressed lncRNAs between 32 TNBC cell lines and 30 non-TNBC cell lines in CCLE database. **C.** Venn diagram of the 308 lncRNAs incorporated into study. **D.** Schematic representation of the CRISPR-Cas9 screening for the determinants of PARPi response in BRCA wild-type MDA-MB-231 cell. **E.** Scatterplot displayed the results of the CRISPR-Cas9 screening.

Firstly, differentially expressed lncRNAs were identified by analyzing the TCGA database, which included 158 TNBC and 14 adjacent normal tissue samples^33^. The results showed that 1873 lncRNAs (fold change ≥ 1.5, *P* value < 0.05) were overexpressed in TNBC tissues compared to adjacent normal tissues (Figure 1A). Next, we analyzed the CCLE database, which contained 32 TNBC cell lines and 30 non-TNBC cell lines (Supplementary Table S2), to identify differentially expressed lncRNAs. Compared to non-TNBC cell lines, a total of 1461 elevated lncRNAs (fold change ≥ 1.5, *P* value < 0.05) were observed in TNBC cell lines (Figure 1B). Subsequently, lncRNAs with higher expression in TNBC from both the TCGA and CCLE databases were intersected, and those lncRNAs reported in the literature associated with DNA damage were retrieved. Finally, a total of 308 lncRNAs were incorporated into this study (Figure 1C).

To investigate whether any of these lncRNAs might govern PARPi response in BRCA wild-type TNBC, we designed and synthesized a sgRNA library targeting 238 out of 308 lncRNAs containing 3706 sgRNAs through employing the genome-wide CRISPR-cas9 knockout library developed by Wei group^28^. Lentiviral vectors were constructed to package the sgRNA library plasmids, which were then transfected into BRCA wild-type MDA-MB-231 cells and divided into PARPi Olaparib (6 μM) and DMSO treated arms. After 14 days of CRISPR-cas9 loss-of-function screening, surviving MDA-MB-231 cells from both treatment arms were harvested for sgRNA sequencing analysis using the Model-based Analysis of Genome-wide CRISPR-cas9 Knockout (MAGeCK) algorithm^30^ (Figure 1D). Compared to the DMSO treated arm, sgRNAs dropped out in the Olaparib treated arm generated a list of lncRNAs, whose knockout sensitized BRCA wild-type TNBC cells to PARPi treatment (Figure 1E). Among the top hits from this screening, the lncRNA MTUS2-AS1 was identified as an essential contributor to the insensitivity of BRCA wild-type TNBC cells to PARPi treatment.

### MTUS2-AS1 was overexpressed in TNBC and associated with Olaparib resistance

**Figure 2:**
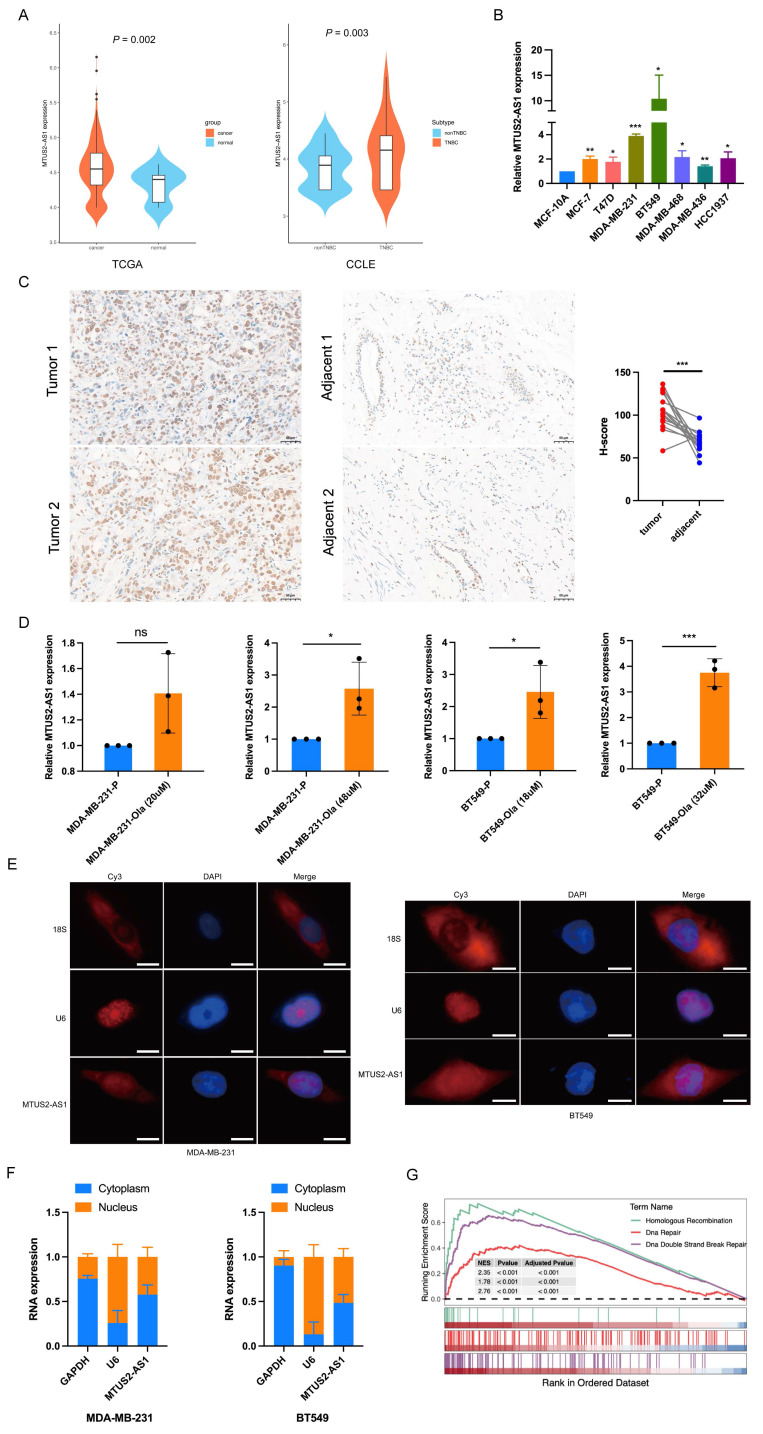
** MTUS2-AS1 was overexpressed in TNBC and associated with Olaparib resistance. A.** The expression of MTUS2-AS1 in TNBC tissues and cell lines. Data from TCGA and CCLE database, respectively.** B.** The expression of MTUS2-AS1 in normal mammary epithelial cell line MCF-10A and BC cell lines from our laboratory. **C.** The expression levels of MTUS2-AS1 in 15 pairs of TNBC tissues and their adjacent normal tissues. **D.** The expression levels of MTUS2-AS1 in the parental and resistant cell lines of MDA-MB-231 and BT549. The cellular location of MTUS2-AS1 in MDA-MB-231 and BT549 cell lines were detected by **E.** fluorescence *in situ* hybridization (FISH) and **F.** nuclear-cytoplasmic fractionation experiments. **G.** MTUS2-AS1 involved in the pathways were analyzed by GSEA. Data are from three independent experiments and shown as mean ± SD. *P* values are from unpaired two-sided Student's* t*-test (A, D), ANOVA test (B) and paired two-sided Student's* t*-test (C). ^*^ *P* < 0.05, ^**^ *P* < 0.01, ^***^ *P* < 0.001, ns, not significant.

Previous bioinformatics analysis showed that the lncRNA MTUS2-AS1 was upregulated in both TNBC tissues and cell lines (Figure 2A). We further examined MTUS2-AS1 expression in the normal mammary epithelial cell line MCF-10A and BC cell lines (MCF-7, T47D, MDA-MB-231, BT549, MDA-MB-468, MDA-MB-436 and HCC1937) by qPCR method. The results revealed that the level of MTUS2-AS1 was elevated in BC cell lines, with significantly higher expression in TNBC cell lines (MDA-MB-231 and BT549) compared to non-TNBC cell lines (MCF-7 and T47D) (Figure 2B). Although no statistically significant differences were observed in MTUS2-AS1 expression in MDA-MB-468 and HCC1937 cell lines compared to non-TNBC cell lines, a trend toward increased expression was noted. Indeed, MTUS2-AS1 expression in MDA-MB-436 cells seems to be lower than that in MCF-7 and T47D cells. Collectively, these findings suggest MTUS2-AS1 exhibits high expression levels across the majority of TNBC cell lines. Further validation was conducted in 15 pairs of TNBC tissues and their adjacent normal tissues (Figure 2C). Additionally, olaparib-resistant MDA-MB-231 and BT549 cells were generated using a low-dose intermittent stimulation method. The resistance index of MDA-MB-231 cells was 3.16 and 3.6 at Olaparib concentrations of 20 μM and 46 μM, respectively. Similarly, the resistance index of BT549 cells was 3.75 and 7.37 at Olaparib concentrations of 18 μM and 32 μM, respectively (Supplementary Figure S1A, B). Furthermore, the expression levels of MTUS2-AS1 in the parental and resistant cell lines of MDA-MB-231 and BT549 were measured by qPCR. An increasing expression of MTUS2-AS1 with the degree of drug resistance in TNBC cells were observed (Figure 2D), indicating that MTUS2-AS1 might confer Olaparib resistance to TNBC cells. Afterwards, fluorescence *in situ* hybridization (FISH) and nuclear-cytoplasmic fractionation experiments showed that MTUS2-AS1 was localized in both the nucleus and cytoplasm of TNBC cells (Figure 2E, F). Gene Set Enrichment Analysis (GSEA) results suggested that the expression level of MTUS2-AS1 was closely associated with pathways related to DNA repair, homologous recombination, and DNA double-strand break repair (Figure 2G), all of which have been linked to PARPi resistance.

### MTUS2-AS1 knockdown suppressed DNA damage repair and enhanced the anticancer effects of PARPi in BRCA-wild TNBC cells

**Figure 3:**
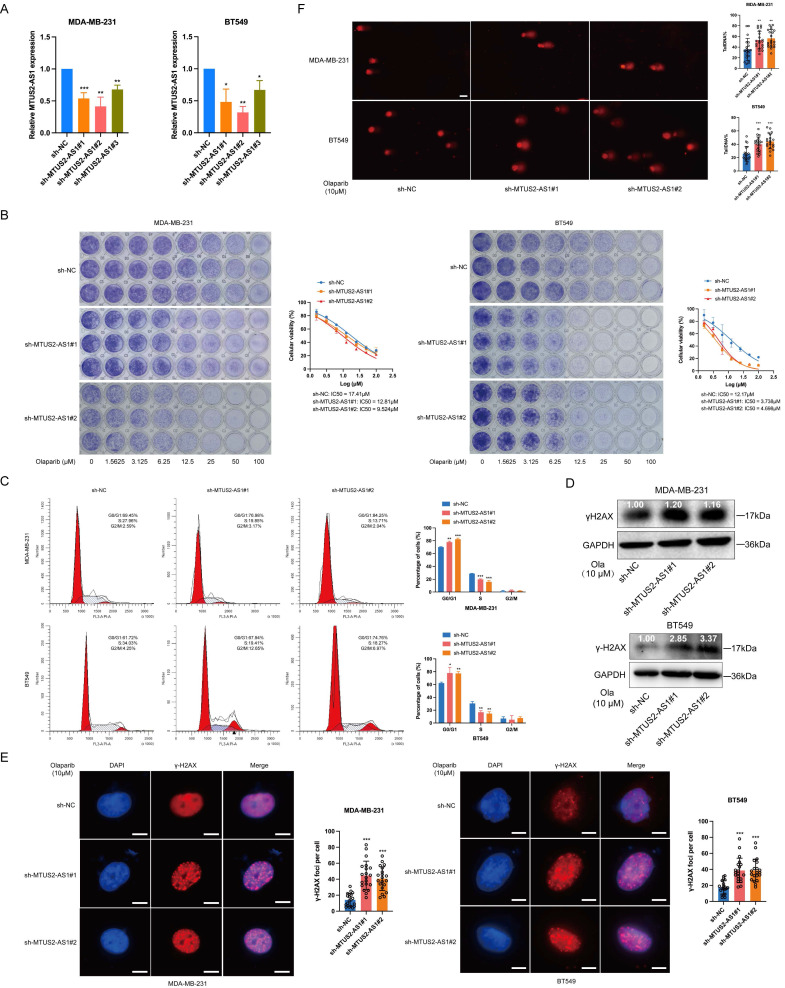
** MTUS2-AS1 knockdown suppressed DNA damage repair and enhanced the anticancer effects of PARPi in BRCA-wild TNBC cells. A.** The knockdown efficiency of shRNA-targeting MTUS2-AS1 in MDA-MB-231 and BT549 cell lines. **B.** The IC50 value of Olaparib in the negative control group and the MTUS2-AS1 knockdown group. **C.** The proliferation ability was assessed by cell cycle assays in MDA-MB-231 and BT549 cells transfected with sh-NC and sh-MTUS2-AS1 group under Olaparib (10μM/3 days) treatment. The level of γH2AX was detected by **D.** western blotting, and **E.** immunofluorescence staining in MTUS2-AS1 knockdown cells compared to control cells under Olaparib (10μM/3 days) treatment. **F.** The impact of MTUS2-AS1 expression on DNA repair activity in Olaparib (10μM/3 days) treatment induced DNA damage was evaluated by comet assays in MDA-MB-231 and BT549 cells. Data are from three independent experiments and shown as mean ± SD. *P* values are from ANOVA test (A, C, E, F). ^*^ *P* < 0.05, ^**^ *P* < 0.01, ^***^ *P* < 0.001.

To determine whether MTUS2-AS1 knockdown might enhance the sensitivity of BRCA wild-type TNBC cells to PARPi treatment, we designed three lentiviral vectors expressing shRNAs targeting MTUS2-AS1 (sh-MTUS2-AS1#1, #2 and #3) and transfected them into BRCA wild-type MDA-MB-231 and BT549 cells to reduce MTUS2-AS1 expression (Supplementary Figure S2A, B). The qPCR results showed that the knockdown efficiency of sh-MTUS2-AS1#1 and #2 were the most significant compared to shRNA negative control (sh-NC) group (Figure 3A). Therefore, the lentiviral vectors containing sh-MTUS2-AS1#1 and #2 were employed to silence MTUS2-AS1 expression in our subsequent experiments.

As mentioned above, PARPi exert efficacy in cancers with BRCA mutations. We firstly measured the half maximal inhibitory concentration (IC50) of Olaparib in BRCA1-knockout TNBC cells. The results showed that the IC50 values of MDA-MB-231 and BT549 cells were lower in the BRCA1-knockout group (Supplementary Figure S3A, B). Under the same experimental conditions, we calculated the IC50 values in the negative control group and the MTUS2-AS1 knockdown group. Notably, the cell viability also decreased in the MTUS2-AS1 knockdown group compared to the negative control group (Figure 3B). Additionally, cell cycle assays revealed that TNBC cells with MTUS2-AS1 knockdown exhibited a significant reduction in S phase under Olaparib (10μM/3 days) treatment (Figure 3C), indicating an inhibitory effect of cell proliferation. This phenomenon further supported that MTUS2-AS1 knockdown sensitized BRCA wild-type TNBC cells to PARPi Olaparib. Furthermore, we evaluated the proliferation ability of TNBC cells under MTUS2-AS1 knockdown without Olaparib treatment. The results showed that DNA synthesis in TNBC cells exhibited a decreasing trend upon silencing MTUS2-AS1, suggesting that MTUS2-AS1 knockdown may suppress TNBC cells proliferation to a certain extent (Supplementary Figure S3C).

To explore whether MTUS2-AS1 influences the efficacy of PARPi by modulating DNA damage repair, we assessed the formation of phospho-H2AX (γH2AX) foci in TNBC cells, a well-established marker for DSBs. Western blotting results showed that the level of γH2AX was elevated in MTUS2-AS1 knockdown cells compared to control cells under Olaparib (10μM/3 days) treatment (Figure 3D), suggesting increased DNA damage after Olaparib treatment with MTUS2-AS1 knockdown. Immunofluorescence staining further supported these findings (Figure 3E). Moreover, comet assays were performed to evaluate the effect of MTUS2-AS1 expression on DNA repair activity after Olaparib (10μM/3 days) treatment induced DNA damage. As shown in the Figure 3F, the comet tail length was longer in MTUS2-AS1 knockdown cells than in the control group, indicating DNA repair activity was attenuated in TNBC cells with MTUS2-AS1 knockdown under Olaparib treatment.

**Figure 4:**
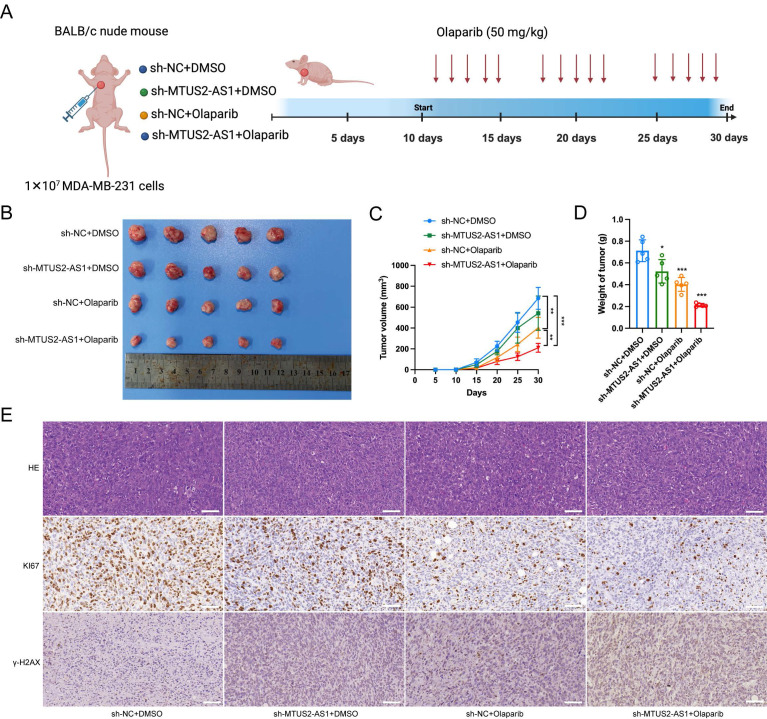
** Xenograft tumor models were utilized to assess the efficacy of MTUS2-AS1 knockdown and PARPi treatment *in vivo*. A.** Schematic model for tumor xenograft and Olaparib treatment. **B.** Representative images of the tumor xenograft. **C.** Tumor volume and **D.** weight were measured in each group. **E.** Representative HE and IHC images revealed the expression of KI67 and γH2AX in each group. Data are from at least three independent experiments and shown as mean ± SD. *P* values are from ANOVA test (C, D). ^*^ *P* < 0.05, ^**^ *P* < 0.01, ^***^ *P* < 0.001.

Next, xenograft tumor models were used to evaluate the efficacy of MTUS2-AS1 knockdown combined with PARPi treatment *in vivo*. As shown in the Figure 3A, the knockdown efficiency of sh-MTUS2-AS1#2 was more significant than that of sh-MTUS2-AS1#1. Therefore, we established xenograft tumor models using MDA-MB-231 cells expressing sh-MTUS2-AS1#2 or sh-NC. The results showed that the volume and weight of xenograft tumor in nude mice were markerly decreased in the MTUS2-AS1 knockdown group compared to the control group under Olaparib treatment (Figure 4A-D). Additionally, IHC staining revealed that xenograft tumors combined with MTUS2-AS1 knockdown and PARPi treatment exhibited lower KI67 levels and higher γH2AX expression than those in the control group (Figure 4E). Together, these findings indicated that MTUS2-AS1 knockdown impaired DNA damage repair, thereby enhancing the anticancer efficacy of PARPi in BRCA-wild TNBC cells.

### MTUS2-AS1 knockdown accelerated DDX5 protein degradation through ubiquitin/proteasome-dependent pathway

**Figure 5:**
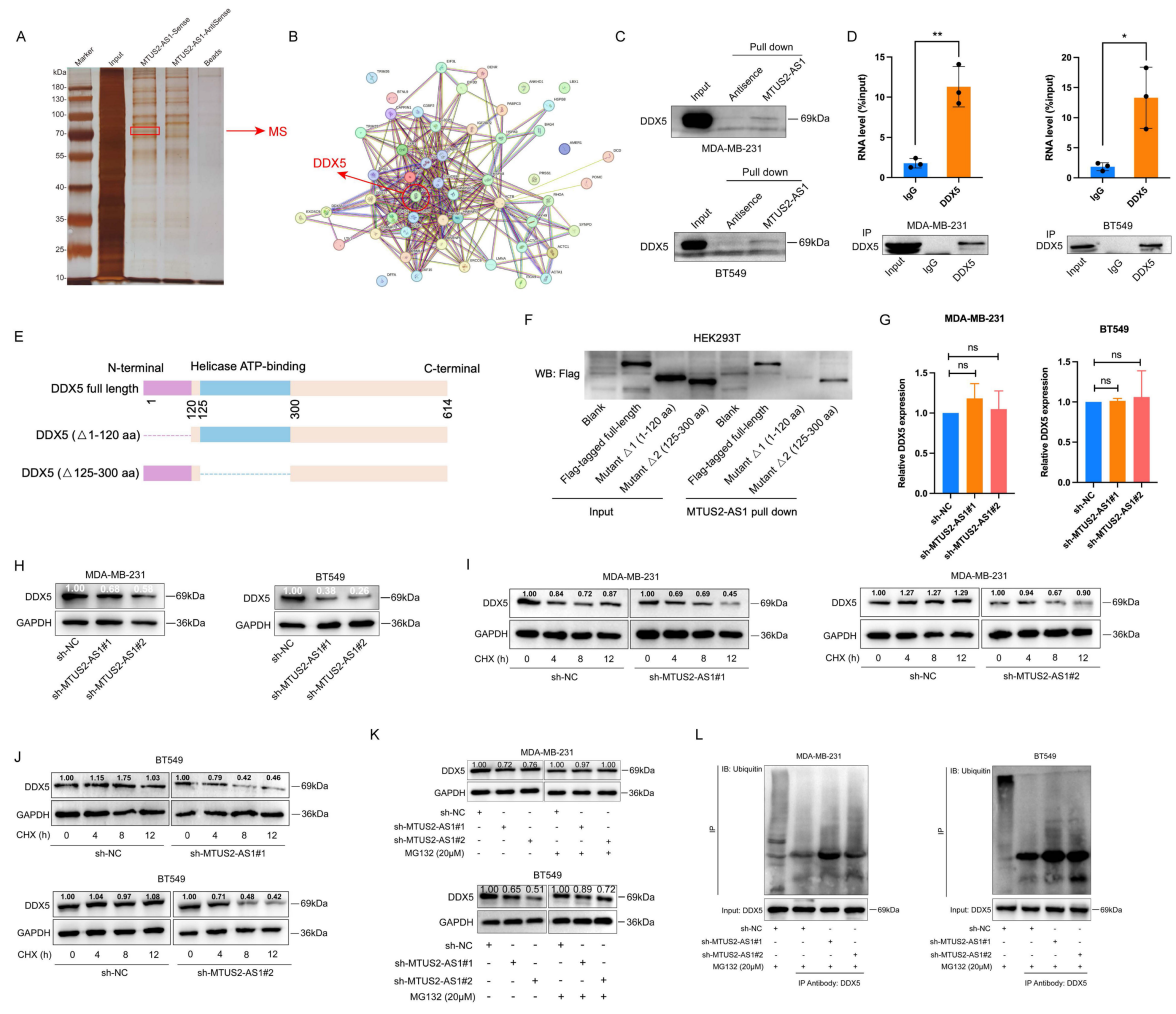
** MTUS2-AS1 knockdown accelerated DDX5 protein degradation through ubiquitin/proteasome-dependent pathway. A.** RNA pull-down combined with mass spectrometry analysis identified proteins interacted with MTUS2-AS1. **B.** Protein-protein interaction networks (PPI) showed the interaction among the proteins pulled down by MTUS2-AS1. **C.** DDX5 protein interacted with MTUS2-AS1 in MDA-MB-231 and BT549 cells was verified by western blotting. **D.** The interaction between MTUS2-AS1 and DDX5 in MDA-MB-231 and BT549 cells was validated by RIP assays. **E.** Flag-tagged DDX5 full-length expression plasmid and two truncated DDX5 mutants (deletion of residues △1 1-120 aa and helicase ATP-binding domain △2 125-300 aa) were designed. **F.** RNA pull down assays and western blotting were performed to validate the specific binding regions between MTUS2-AS1 and DDX5. The expression of DDX5 was evaluated in MDA-MB-231 and BT549 cells with MTUS2-AS1 knockdown by **G.** qPCR and **H.** western blotting. The expression of DDX5 protein was measured under the protein synthesis inhibitor cycloheximide (CHX) treatment in **I.** MDA-MB-231 and **J.** BT549 cell lines. **K.** The expression of DDX5 protein was detected under MG132 treatment in MDA-MB-231 and BT549 cells with MTUS2-AS1 knockdown. **L.** The ubiquitination level of DDX5 protein in MDA-MB-231 and BT549 cells with different MTUS2-AS1 expression was examined by western blotting. Data are from three independent experiments and shown as mean ± SD. *P* values are from unpaired two-sided Student's* t*-test (D) and ANOVA test (G). ^*^ *P* < 0.05, ^**^ *P* < 0.01, ns, not significant.

Currently, accumulating evidence has demonstrated that lncRNAs can exert their biological functions through interactions with RNA binding proteins^34^, ^35^. To elucidate the underlying mechanism by which MTUS2-AS1 regulates DNA damage repair, an amount of proteins interacting with MTUS2-AS1 were identified using RNA pull down combined with mass spectrometry analysis (Supplementary Table S3). Protein-protein interaction (PPI) network analysis revealed that DEAD-box RNA helicase DDX5, which exhibited a high interaction score, was the core node among these binding proteins (Figure 5A, B). Therefore, our subsequent experiments focused on the DDX5 protein. Firstly, RNA pull down followed by western blotting confirmed that DDX5 protein could interact with MTUS2-AS1 in MDA-MB-231 and BT549 cells (Figure 5C). Secondly, RIP assays displayed the enrichment of MTUS2-AS1 in RNA-protein complexes precipitated by an anti-DDX5 antibody, further validating the interaction between MTUS2-AS1 and DDX5 in TNBC cells (Figure 5D). Additionally, to identify the specific binding regions of MTUS2-AS1 that interact with DDX5, we predicted the potential binding sites between MTUS2-AS1 and DDX5 using HDOCK SERVER (Supplementary Figure S4A) and designed Flag-tagged DDX5 full-length expression plasmid and two truncated DDX5 mutants (deletion of residues △1 1-120 aa and helicase ATP-binding domain △2 125-300 aa) (Figure 5E). Serial plasmids were transfected into HEK293T cells, followed by RNA pull down assays. Western blotting showed that △1 mutant abolished its interaction with MTUS2-AS1, indicating that △1 mutant is essential for its interaction with MTUS2-AS1 (Figure 5F). Furthermore, to evaluate whether MTUS2-AS1 could affect the expression of DDX5, qPCR and western blotting were performed. The results demonstrated that MTUS2-AS1 knockdown significantly suppressed DDX5 protein expression in both MDA-MB-231 and BT549 cells, whereas had no influence on the mRNA levels of DDX5 (Figure 5G, H).

Subsequently, to illustrate the role of MTUS2-AS1 in the regulation of DDX5 protein, we assessed the DDX5 protein expression in TNBC cells under the protein synthesis inhibitor cycloheximide (CHX) treatment. The results showed that DDX5 protein expression reduced more in the MTUS2-AS1 knockdown group than in the negative control group (Figure 5I, J). Nevertheless, the inhibitor of 26S protostome MG132 treatment could recue the reduction of DDX5 protein induced by MTUS2-AS1 knockdown in both MDA-MB-231 and BT549 cells (Figure 5K). Furthermore, we examined the ubiquitination level of DDX5 protein in TNBC cells with different MTUS2-AS1 expression and found that the ubiquitin signals of DDX5 protein were markly increased in the MTUS2-AS1 knockdown group compared to the negative control group (Figure 5L). Collectively, these data suggested that MTUS2-AS1 knockdown might accelerate DDX5 protein degradation through ubiquitin/proteasome-dependent pathway.

### MTUS2-AS1 knockdown induced R-loop accumulation and DNA damage by promoting DDX5 protein degradation

**Figure 6:**
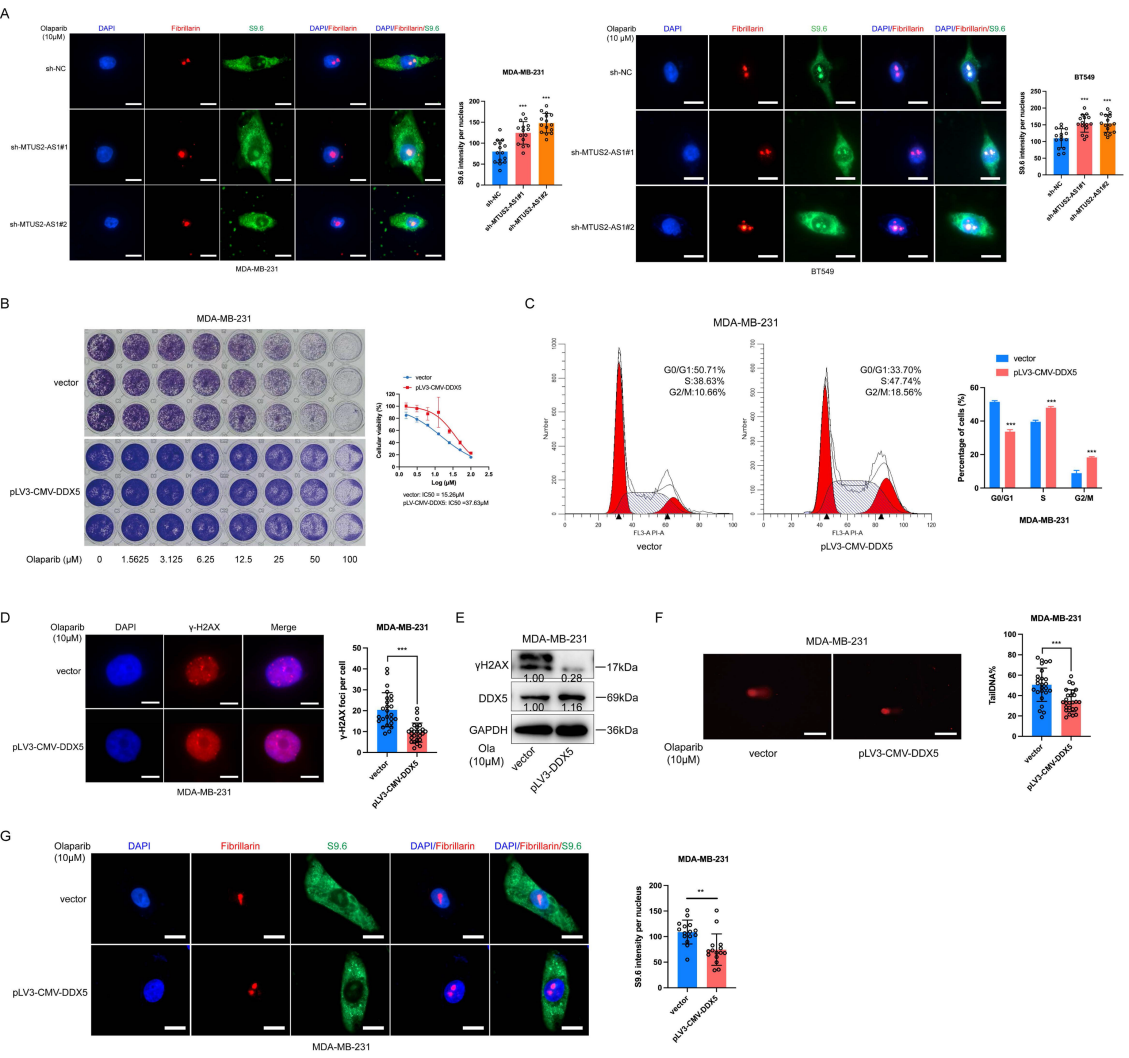
** DDX5 plays important roles in the regulation of DNA damage repair. A.** The nuclear signal intensity of R-loop in MDA-MB-231 and BT549 cells transfected with sh-NC and sh-MTUS2-AS1 group. The cell viability, γH2AX levels, comet tail length and nuclear signal intensity of S9.6 were evaluated in MDA-MB-231 cells transfected with the scramble control and DDX5 overexpression vector by **B.** IC50 assay, **C.** cell cycle, **D.** immunofluorescence staining, **E.** western blotting, **F.** comet assays, and **G.** R-loop immunofluorescence assays. Data are from three independent experiments and shown as mean ± SD. *P* values are from ANOVA test (A) and unpaired two-sided Student's* t*-test (C, D, F, G). ^**^ *P* < 0.01, ^***^ *P* < 0.001.

As we known, DNA-RNA hybrids are generated by nascent RNA and template DNA during DNA transcription, replication, and repair. These hybrids can displace one strand of double-stranded DNA, forming a three-stranded structure known as an R-loop with the remaining DNA strand^36^. Aberrant accumulation of R-loops causes catastrophic replication conflicts and aggravates DNA damage^22^. DDX5 protein has been identified the roles in the resolution of R-loop to prevent cell death caused by DNA damage^19^, ^37^. Previously, we demonstrated that MTUS2-AS1 could stabilize DDX5 protein to upregulate its expression. To further explore whether MTUS2-AS1 might act as a regulator for R-loop, we performed immunofluorescent staining to detect the expression of R-loop with the broadly used monoclonal antibody S9.6^38^. Compared with the negative control group, increased nuclear signal intensity of R-loop was observed upon MTUS2-AS1 knockdown in both MDA-MB-231 and BT549 cells (Figure 6A), suggesting aggravated DNA damage. This result was consistent with our prior findings of the elevated γH2AX levels in MTUS2-AS1 knockdown cells (Figure 3D, E). To confirm the nuclear S9.6 signals originated from the R-loop, we pretreated cells with RNase H, a nuclease that eliminates R-loop signals through degrading the RNA portion of R-loop^39^. The results showed that RNase H treatment removed the nuclear signal intensity of R-loop upon MTUS2-AS1 knockdown in TNBC cells (Supplementary Figure S4B), confirming these nuclear S9.6 signals were indeed derived from the R-loop. Additionally, we constructed an overexpression vector for DDX5 in MDA-MB-231 cells to assess the function of DDX5 in the regulation of DNA damage repair (Supplementary Figure S4C). Unsurprisingly, compared with the scramble control, DDX5 overexpression promoted cell proliferation, decreased γH2AX levels, shortened comet tail length and diminished nuclear signal intensity of S9.6 (Figure 6B-G), indicating DNA damage was inhibited.

**Figure 7:**
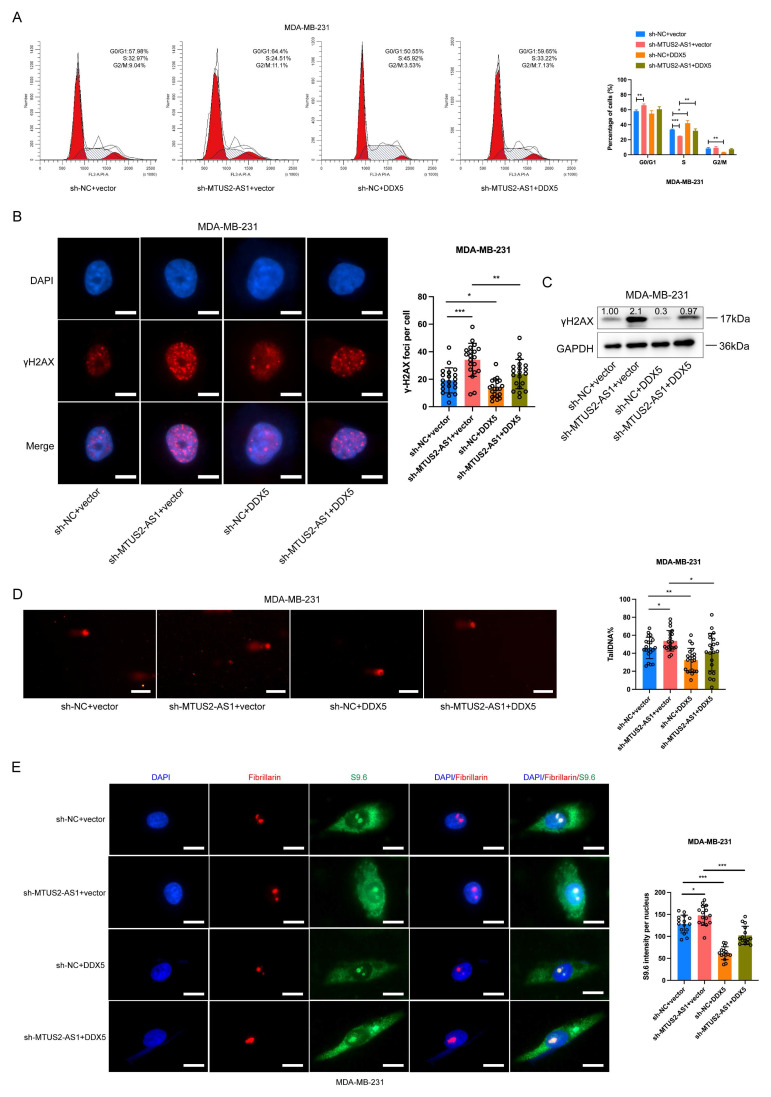
** MTUS2-AS1 knockdown induced R-loop accumulation and DNA damage by promoting DDX5 protein degradation.** Rescue experiments performed in MDA-MB-231 cells transfected with sh-MTUS2-AS1 and DDX5 overexpression plasmid by **A.** cell cycle, **B.** immunofluorescence staining, **C.** western blotting, **D.** comet assays, and **E.** R-loop immunofluorescence assays. Data are from three independent experiments and shown as mean ± SD. *P* values are from ANOVA test (A, B, D, E). ^*^ *P* < 0.05, ^**^ *P* < 0.01, ^***^ *P* < 0.001.

Next, to comfirm whether MTUS2-AS1 exerts its function through interaction with DDX5, we successfully transfected the DDX5 overexpression plasmid into the MDA-MB-231 cells with MTUS2-AS1 knockdown and then performed rescue experiments (Supplementary Figure S4D). As expected, DDX5 overexpression could partially reverse the inhibitory effect on cell proliferation, increased γH2AX levels, comet tail length and nuclear signal intensity of S9.6 induced by MTUS2-AS1 knockdown (Figure 7A-E). Additionally, to investigate whether the effect of DDX5 reducing R-loop accumulation depends on its known ATP-dependent RNA helicase activity. We transfected the helicase-dead mutant of DDX5 (△2 125-300 aa) into MTUS2-AS1 knockdown cells and found that this mutant failed to reverse the increase of nuclear S9.6 signal intensity induced by MTUS2-AS1 knockdown (Supplementary Figure S4E). Collectively, these results demonstrated that MTUS2-AS1 knockdown promoted R-loop accumulation and DNA damage by accelerating DDX5 protein degradation.

## Discussion

**Figure 8:**
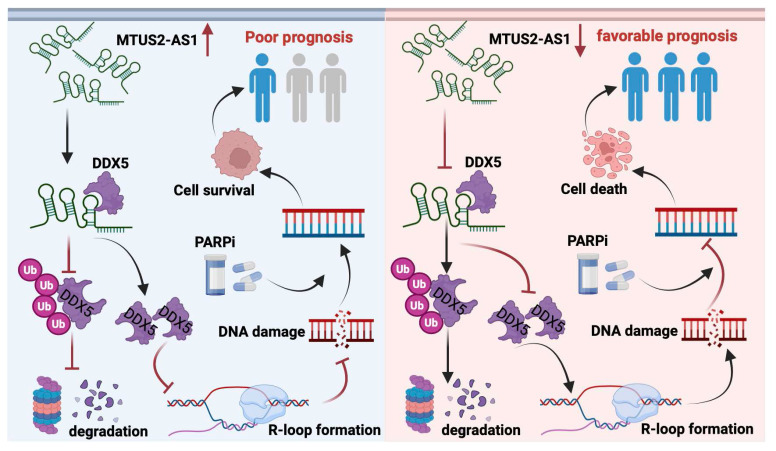
** Schematic model of lncRNA MTUS2-AS1 as an essential contributor to the insensitivity of BRCA wild-type TNBC cells to PARPi treatment.** MTUS2-AS1 upregulates DDX5 protein expression by maintaining its stability, thereby promoting R-loop resolution, and suppressing DNA damage. MTUS2-AS1 knockdown accelerates DDX5 protein degradation, reduces DDX5 protein expression, inhibits R-loop resolution, promotes DNA damage, and ultimately enhances the PARPi sensitivity in TNBC cells.

In recent years, accumulating evidence has demonstrated that PARPi showed effective in patients with wild-type BRCA^40^, suggesting their potential applicability to TNBC patients lacking BRCA mutations. However, the key molecules and mechanisms influencing the sensitivity of PARPi treatment remain poorly understood. In our study, we identified lncRNA MTUS2-AS1 as an essential contributor to the insensitivity of BRCA wild-type TNBC cells to PARPi treatment. Mechanistically, MTUS2-AS1 upregulates DDX5 protein expression by maintaining its stability, thereby promoting R-loop resolution, and suppressing DNA damage. MTUS2-AS1 knockdown accelerates DDX5 protein degradation, reduces DDX5 protein expression, inhibits R-loop resolution, promotes DNA damage, and ultimately enhances the PARPi sensitivity in TNBC cells (Figure 8).

At present, CRISPR-Cas9 screening technology is widely employed as an unbiased approach to identify key determinants in various biological processes, particularly for understanding protein-coding genes. Moldovan *et al*. identified the ubiquitin ligase HUWE1 and the histone acetyltransferase KAT5 as the regulators of PARPi response through complementary CRISPR knockout and activation screens^41^. However, apart from protein-coding genes, the vast majority of the human genome consists of non-coding RNAs (ncRNAs)^42^. Among these, a large number of lncRNAs loci have been annotated, inferring their as-yet-undefined roles in modulating the sensitivity of drugs. Tassone *et al*.^43^ unveiled that RP11-350G8.5 emerged as the most essential gene and a promising target in multiple myeloma cells and their bortezomib-resistant derivative using a CRISPR-Cas9 strategy previously developed by the Wei group^44^, which utilizing paired-gRNAs (pgRNAs) to produce large-fragment deletions and identify functional lncRNAs. Subsequent studies revealed the pgRNAs-based strategy was cumbersome and complicated, limiting its broader application. Afterwards, the Wei group exploited a genome-wide screening for functional lncRNAs by using Cas9 to target splice sites, thereby achieving exon skipping or intron retention^28^. Our study employed this strategy and identified lncRNA MTUS2-AS1 as an essential contributor to the insensitivity of BRCA wild-type TNBC cells to PARPi treatment.

MTUS2-AS1 is termed as microtubule associated scaffold protein 2 (MTUS2) antisense RNA 1, but with fewer investigations. Our study revealed that MTUS2-AS1 was overexpressed in TNBC tissues compared to adjacent normal tissues through TCGA database analysis and clinical TNBC samples validation. Also, the elevated expression of MTUS2-AS1 was observed in TNBC cell lines compared to non-TNBC cell lines using CCLE database. Additionally, we detected the expression of MTUS2-AS1 in normal mammary epithelial cell line MCF-10A and BC cell lines (MCF-7, T47D, MDA-MB-231, BT549, MDA-MB-468, MDA-MB-436 and HCC1937) and found that the level of MTUS2-AS1 was elevated in BC cell lines, with notably high expression in the majority of TNBC cell lines. These findings confirmed MTUS2-AS1 was overexpressed in TNBC.

Numerous studies have reported that the efficacy of drugs can be modulated by the abnormal expression of lncRNAs^45^. For instance, Slack *et al*. characterized a lncRNA named TRIDENT, which is induced upon epithelial growth factor receptor (EGFR) activation in non-small cell lung cancer. Knockdown of TRIDENT leads to the accumulation of DNA damage in cancer cells via decreased TRIM28 phosphorylation, thereby sensitizing the cells to chemotherapeutic drugs^46^. Guan *et al*. demonstrated that high expression of the lncRNA GDIL serves as a scaffold for XRN2 to recognize and degrade CHAC1 mRNA, leading to glutathione (GSH) accumulation by inhibiting GSH degradation and contributing to hyposensitivity to platinum-based therapy. Suppression of GDIL restored drug sensitivity in platinum-resistant cell lines and patient-derived xenografts^47^. In our research, we identified MTUS2-AS1 as an essential contributor to the insensitivity of BRCA wild-type TNBC cells to PARPi treatment through CRISPR-cas9 loss-of-function screening. Additionally, an increasing expression of MTUS2-AS1 with the degree of drug resistance in TNBC cells were observed. Further analysis of public database showed that the expression level of MTUS2-AS1 was closely associated with pathways involved in DNA repair, homologous recombination, and DNA double-strand break repair. These results provide indirect evidence linking MTUS2-AS1 to PARPi sensitivity. Subsequent functional experiments, including IC50 detection, cell cycle assays, immunofluorescence staining of γH2AX foci, comet assays, and xenograft tumor models indicated MTUS2-AS1 might influence the efficacy of PARPi by regulating DNA damage repair.

As is well known, the biological functions of lncRNA depend on its subcellular localization^48^. Our study showed that MTUS2-AS1 was localized in both the nucleus and cytoplasm of TNBC cells through FISH and nuclear-cytoplasmic fractionation experiments. Alajez *et al*. reviewed that lncRNA, whether derived from nucleus or cytoplasm, could interact with RNA binding proteins, and play pivotal roles in key regulatory mechanisms across various cellular processes^49^. We identified a number of proteins interacting with MTUS2-AS1 through RNA pull down combined with mass spectrometry and clarified the crucial role of DDX5 protein by PPI analysis. Increasing evidence indicated that DDX5 was involved in multiple oncogenic signaling pathways via interactions with different types of molecules. Tong *et al*. found that lncRNA NHEG1 bound to and stabilized DDX5 protein through repressing proteasome-mediated degradation, resulting in β-catenin transactivation and altered expression of target gene associated with neuroblastoma progression^50^. Xie *et al*. demonstrated that AURKAIP1 directly interacted with and stabilized DDX5 protein by preventing its ubiquitination and degradation, and DDX5 overexpression enhanced the activity of Wnt/β-catenin signaling pathway involved in TNBC progression^51^. In our study, we validated the interaction between MTUS2-AS1 and DDX5 in TNBC cells through RNA pull down and RIP assays. Truncation mutation experiments showed that a DDX5 mutant (deletion of residues △1 1-120 aa) is essential for its interaction with MTUS2-AS1. Subsequent experiments, including CHX, MG132 and IP assays suggested that MTUS2-AS1 may stabilize DDX5 protein expression through preventing its ubiquitination and degradation.

As a member of the DEAD-box RNA helicase family, DDX5 plays a critical role in regulating the dynamic balance of R-loops^19^. Aberrant accumulation of R-loop leads to genomic instability with DNA damage^52^. Zhou *et al*. demonstrated that lncRNA PLADE binds to and downregulates HNRNPD by VHL-mediated ubiquitination, thereby increasing R-loop levels and DNA damage, which in turn promotes cisplatin sensitivity in high-grade serous ovarian cancer^53^. In our study, increased nuclear signal intensity of R-loop were observed upon MTUS2-AS1 knockdown in both MDA-MB-231 and BT549 cells. RNase H treatment confirmed the specificity of the S9.6 signals. Rescue experiments revealed that DDX5 overexpression partially reversed the increased nuclear signal intensity of S9.6 induced by MTUS2-AS1 knockdown. These results suggested that MTUS2-AS1 knockdown reduces DDX5 protein expression through promoting its ubiquitination and degradation, thereby inhibiting R-loop resolution and aggravating DNA damage, ultimately enhancing the PARPi sensitivity in TNBC cells.

In summary, we identified lncRNA MTUS2-AS1 was significantly correlated with PARPi sensitivity in BRCA wild-type TNBC cells through CRISPR-cas9 loss-of-function screening. *In vitro* and *in vivo* experiments demonstrated that MTUS2-AS1 knockdown suppressed DNA damage repair and enhanced the anticancer effects of PARPi in BRCA-wild TNBC cells. Mechanistically, MTUS2-AS1 upregulates DDX5 protein expression by maintaining its stability, thereby promoting R-loop resolution, and suppressing DNA damage. Knocking down the expression of MTUS2-AS1 accelerates DDX5 protein degradation, reduces DDX5 protein expression, inhibits R-loop resolution, promotes DNA damage, and ultimately enhances the PARPi sensitivity in TNBC cells. Our study provided new insights into exploring the key molecules and mechanisms influencing the sensitivity of PARPi treatment, promoting the optimized use of PARPi in TNBC patients.

## Supplementary Material

Supplementary figures and tables.

## Data Availability

The data that support the findings of this study are available from the corresponding author on reasonable request.
