# Supplementary material for: MTUS2-AS1 suppression promotes DDX5 protein degradation to enhance the sensitivity of PARP inhibitors in BRCA-wild triple negative breast cancer
Source: Int J Biol Sci. 2026 Jul 20;22(12):6735–51. doi: 10.7150/ijbs.133410 (PMC13412408; doi:10.7150/ijbs.133410)
Supplement: Supplementary file 1 — Supplementary figures and tables. [file ijbsv22p6735s1.pdf]

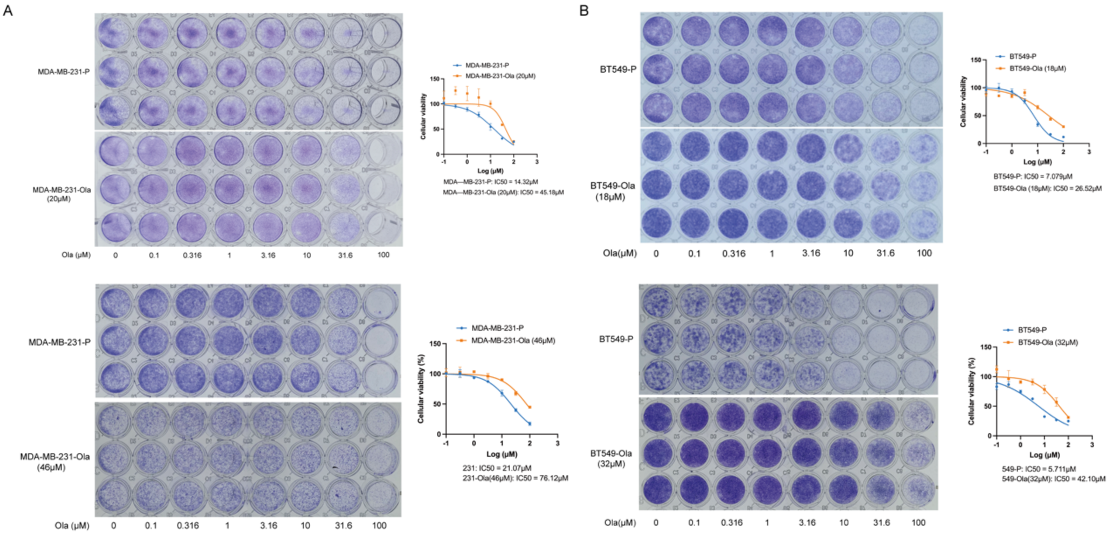

Figure S1 The resistance index of Olaparib-resistant MDA-MB-231 and BT549 cells. The concentrations of Olaparib treatment were (A) 20 μM and 46 μM in MDA-MB-231 cells, (B) 18 μM and 32 μM in BT549 cells.

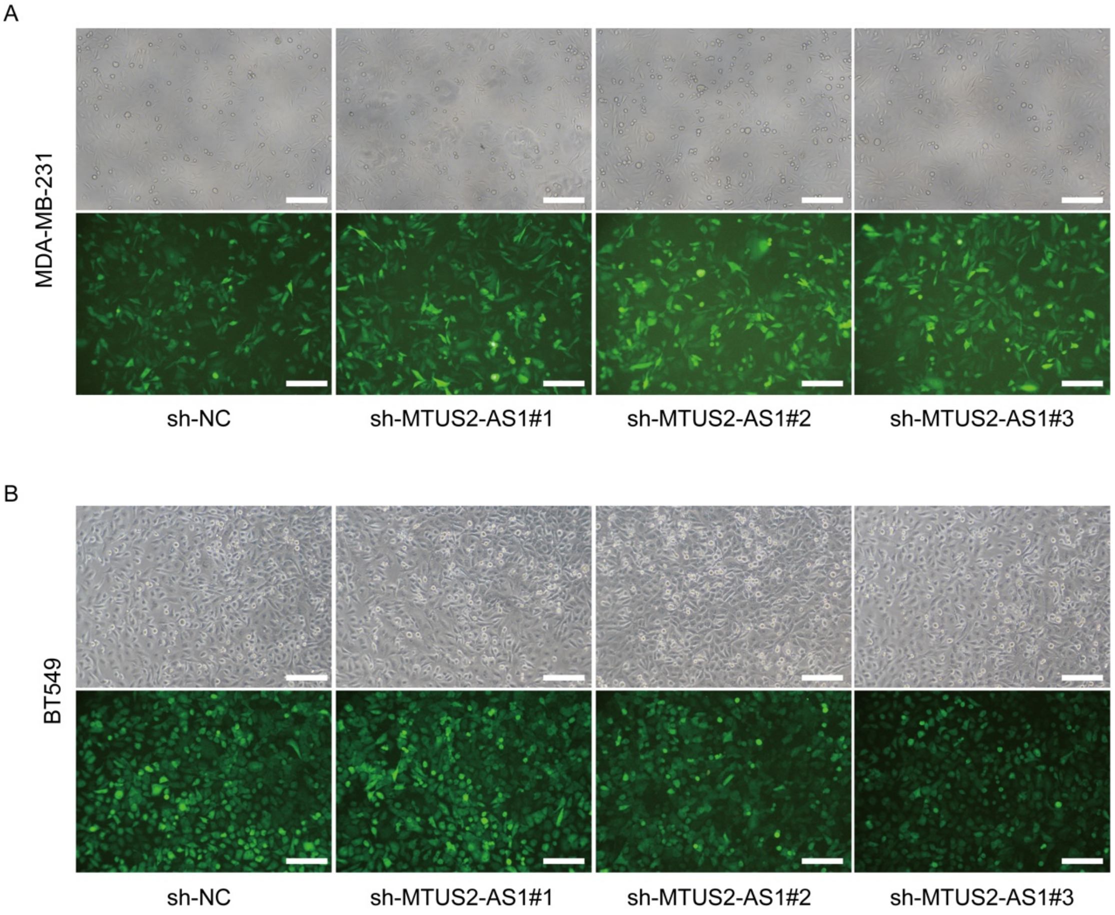

Figure S2 Representative fluorescent images of the knockdown efficiency in (A) MDA-MB-231

and (B) BT549 cells transfected with shRNA-targeting MTUS2-AS1 and negative control.

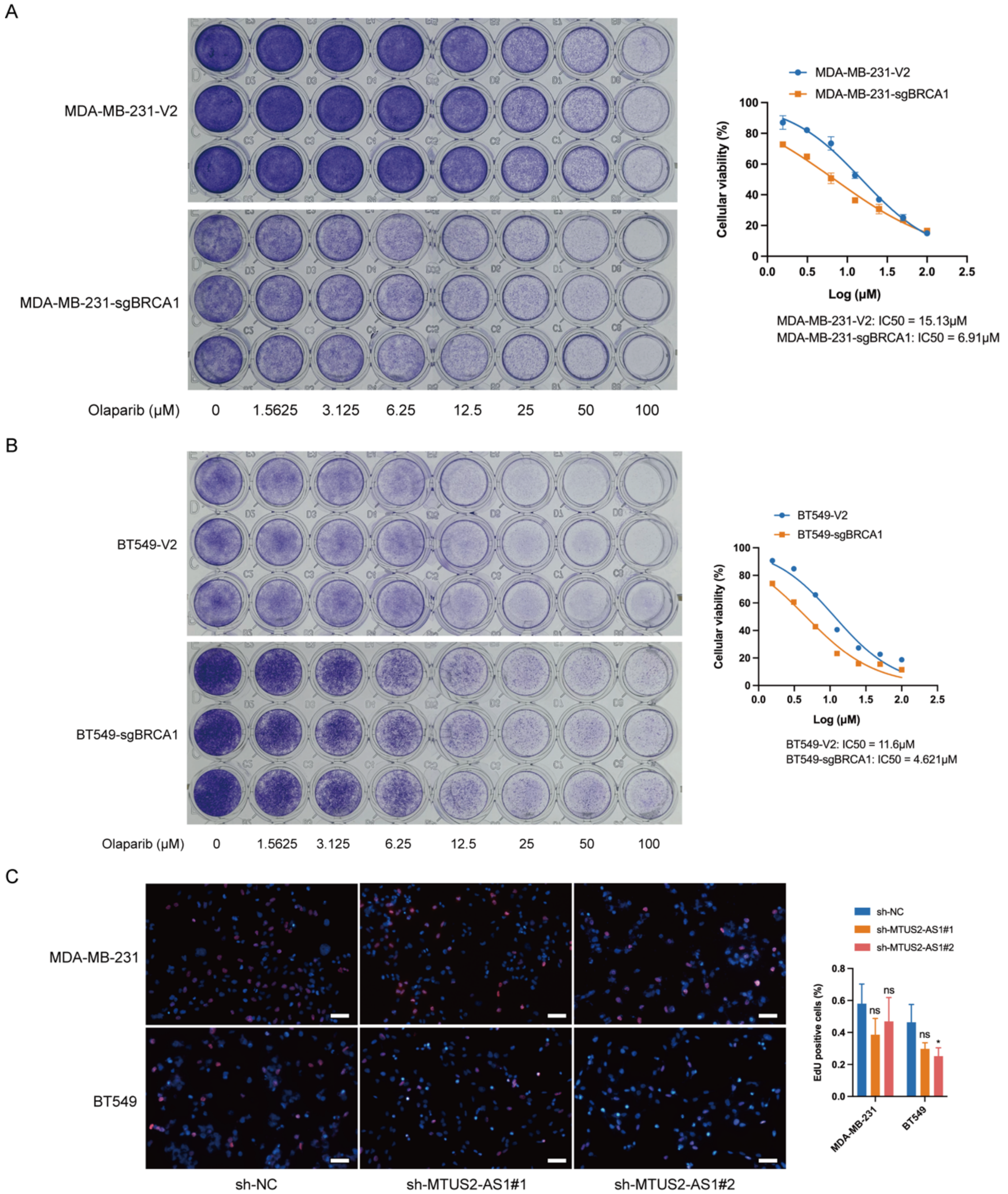

Figure S3 The IC50 value of Olaparib in BRCA1-knockout (A) MDA-MB-231 cells, (B) BT549 cells. (C) The proliferation ability of TNBC cells under MTUS2-AS1 knockdown without Olaparib treatment were performed by EdU assay. Data are from three independent experiments and shown as mean ± SD. *P* values are from ANOVA test (C). $^{*}P < 0.05$, ns, not significant.

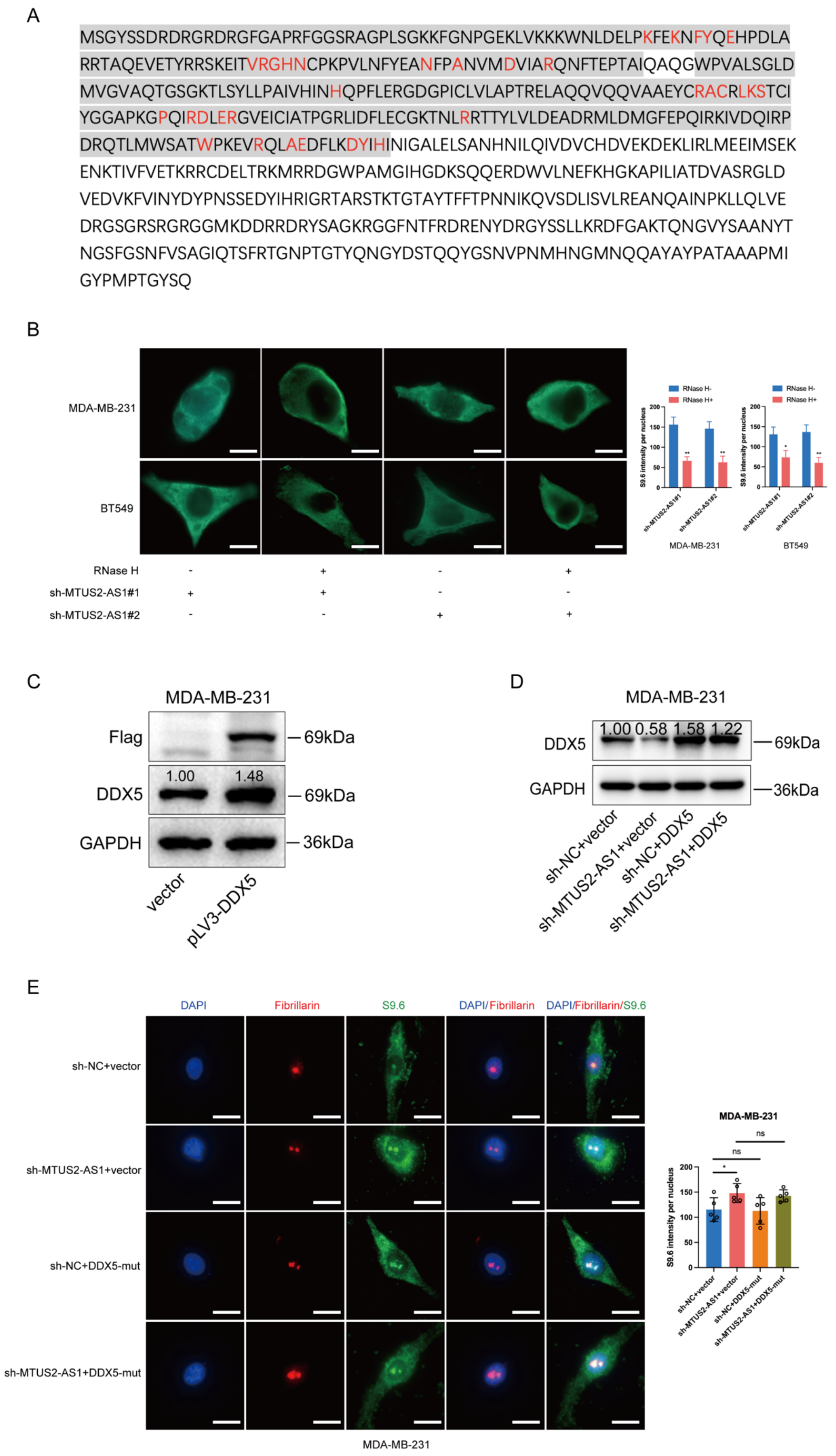

A
MSGYSSDRDRGRDRGFGAPRFGGSRAGPLSGKKFGNPGEKLVKKKWNLDELPKFEKNFYQEHPDLA
RRTAQEVETYRRSKEITVRGHNCPKPVLNFYEANFPANVMDVIARQNFTEPTAIQAQGWPVALSGLD
MVGVAQTGSGKTLSYLLPAIVHINHQPFLERGDGPICLVLAPTRELAQQVQQVAAEYCRACRLKSTCI
YGGAPKGPQIRDLERGVEICIATPGRLIDFLECGKTNLRRTTYLVLDEADRMLDMGFEPQIRKIVDQIRP
DRQTLMWSATWPKEVRQLAEDFLKDYIHINIGALELSANHNILQIVDVCHDVEKDEKLIRLMEEIMSEK
ENKTIVFVETKRRCDELTRKMRRDGWPAMGIHGDKSQQERDWVLNEFKHGKAPILIATDVASRGLD
VEDVKFVINYDYPNSSEDYIHRIGRTARSTKTGTAYTFFTPNNIKQVSDLISVLREANQAINPKLLQLVE
DRGSGRSRGRGGMKDDRRDRYSAGKRGGFNTFRDRENYDRGYSSLLKRDFGAKTQNGVYSAANYT
NGSFGSNFVSAGIQTSFRTGNPTGTYQNGYDSTQQYGSNVPNMHNGMNQQAYAYPATAAAPMI
GYPMPTGYSQ
B
MDA-MB-231
BT549
RNase H
sh-MTUS2-AS1#1
sh-MTUS2-AS1#2
RNase H-
RNase H+
S9.6 intensity per nucleus
MDA-MB-231
BT549
C
MDA-MB-231
Flag
DDX5
GAPDH
1.00
1.48
69kDa
69kDa
36kDa
vector
pLV3-DDX5
D
MDA-MB-231
DDX5
GAPDH
1.00 0.58 1.58 1.22
69kDa
36kDa
sh-NC+vector
sh-MTUS2-AS1+vector
sh-NC+DDX5
sh-MTUS2-AS1+DDX5
E
DAPI
Fibrillarin
S9.6
DAPI/Fibrillarin
DAPI/Fibrillarin/S9.6
sh-NC+vector
sh-MTUS2-AS1+vector
sh-NC+DDX5-mut
sh-MTUS2-AS1+DDX5-mut
MDA-MB-231
MDA-MB-231
S9.6 intensity per nucleus
ns
ns
*

Figure S4 (A) The potential binding sites between MTUS2-AS1 and DDX5 were predicted by HDOCK SERVER. Red amino acid symbol, potential binding sites. Grey region, two truncated DDX5 mutants (deletion of residues △1 1-120 aa and helicase ATP-binding domain △2 125-300 aa). (B) The nuclear signal intensity of R-loop upon MTUS2-AS1 knockdown in MDA-MB-231 and BT549 cells with RNase H treatment. The overexpression efficiency in MDA-MB-231 cells (C) transfected with DDX5 overexpression plasmid, (D) co-transfected with DDX5 overexpression plasmid and sh-MTUS2-AS1. (E) Rescue experiments performed by R-loop immunofluorescence assays in MTUS2-AS1 knockdown cells transfected with the helicase-dead mutant of DDX5 (△125-300 aa) plasmid. Data are from three independent experiments and shown as mean ± SD. *P* values are from unpaired two-sided Student's *t*-test (B) and ANOVA test (E). $^{*}P < 0.05$, $^{**}P < 0.01$, ns, not significant.

Table S1 The sequences of primers used in this study.

Gene	Sense（5'-3'）	Antisense（3'-5'）
GAPDH	GCCGTCTAGAAAAACCTGCC	CCACCTGGTGCTCAGTGTAG
MTUS2-AS1	AACACGGCAGTAGTCCTCGTT	GAGCTTCTTGGTAAACCTGTCTGA
U6	CTCGCTTCGGCAGCACA	AACGCTTCACGAATTTGCGT
DDX5	CTAAGTGGATTGGATATGGTTGGAGTG	TCTAGGAATGGCTGATGATTGATGTG
sh-MTUS2-AS1#1	CTATGAGCGATGACGGCAAAG	
sh-MTUS2-AS1#2	GATGAAATGTTCTGATAATAC	
sh-MTUS2-AS1#3	CACCACATTGTATACTTAAAT	
MTUS2-AS1 probe1	CTTTGCCGTCATCGCTCATAGCG	
MTUS2-AS1 probe2	GAGTCGTGTCACAGGGAACAGCTG	
MTUS2-AS1 probe2	CTGGAGTCCATCGAGTGACAGTTTC	

Table S2 32 TNBC cell lines and 30 non-TNBC cell lines in the CCLE database.

DepMap_ID	cell_line_name	Subtype
ACH-000288	BT-549	TNBC
ACH-001388	SUM-102PT	TNBC
ACH-001389	SUM-1315MO2	TNBC
ACH-001393	SUM-190PT	nonTNBC
ACH-001395	SUM-44PE	nonTNBC
ACH-001397	SUM225CWN	nonTNBC
ACH-001514	HCC1008	nonTNBC
ACH-002324	HCC3153	TNBC
ACH-002327	MCF12A	TNBC
ACH-002328	MX1	TNBC
ACH-000017	SK-BR-3	nonTNBC
ACH-000019	MCF7	nonTNBC
ACH-000044	MDA-MB-134-VI	nonTNBC
ACH-000097	ZR-75-1	nonTNBC
ACH-000111	HCC1187	TNBC
ACH-000117	EFM-192A	nonTNBC
ACH-000147	T-47D	nonTNBC
ACH-000148	Hs 578T	TNBC
ACH-000196	HCC1599	TNBC
ACH-000212	CAL-120	TNBC
ACH-000223	HCC1937	TNBC
ACH-000248	AU565	nonTNBC
ACH-000258	Du4475	TNBC
ACH-000276	HCC38	TNBC
ACH-000277	HCC1419	nonTNBC
ACH-000330	EFM-19	nonTNBC
ACH-000349	HCC1500	nonTNBC
ACH-000352	HCC1428	nonTNBC
ACH-000374	HCC1143	TNBC
ACH-000536	BT-20	TNBC
ACH-000554	UACC-893	nonTNBC
ACH-000568	UACC-812	nonTNBC
ACH-000573	MDA-MB-436	TNBC
ACH-000621	MDA-MB-157	TNBC
ACH-000624	HCC1806	TNBC
ACH-000643	HDQ-P1	TNBC
ACH-000668	HCC70	TNBC
ACH-000691	HCC2157	TNBC
ACH-000699	HCC1395	TNBC

ACH-000711	JIMT-1	nonTNBC
ACH-000721	HMC-1-8	TNBC
ACH-000725	HCC202	nonTNBC
ACH-000755	HCC2218	nonTNBC
ACH-000759	MDA-MB-175-VII	nonTNBC
ACH-000768	MDA-MB-231	TNBC
ACH-000783	CAMA-1	nonTNBC
ACH-000818	BT-483	nonTNBC
ACH-000828	ZR-75-30	nonTNBC
ACH-000849	MDA-MB-468	TNBC
ACH-000856	CAL-51	TNBC
ACH-000857	CAL-85-1	TNBC
ACH-000859	HCC1954	nonTNBC
ACH-000876	MDA-MB-415	nonTNBC
ACH-000902	CAL-148	TNBC
ACH-000910	MDA-MB-453	TNBC
ACH-000927	BT-474	nonTNBC
ACH-000930	HCC1569	nonTNBC
ACH-000934	MDA-MB-361	nonTNBC
ACH-001390	SUM-149PT	TNBC
ACH-001391	SUM-159PT	TNBC
ACH-001392	SUM-185PE	TNBC
ACH-001396	SUM-52PE, SUM52	nonTNBC

Table S3 Mass spectrometry results of RNA pull down assay.

Accession	protein_name	-10LgP	Coverage(%) M35	#Peptides	Area M35	#Unique	#Spec M35	Average Mass
P02768|ALBU_HUMAN	ALBU	281.1451	61.74	35	1.05E+07	35	53	69366.68
P17844|DDX5_HUMAN	DDX5	253.22197	37.13	26	2.43E+06	19	29	69148.08
P04264|K2C1_HUMAN	K2C1	253.14372	40.53	27	4.41E+06	20	28	66038.73
P35908|K22E_HUMAN	K22E	243.09578	37.72	22	1.18E+06	16	25	65432.88
P13645|K1C10_HUMAN	K1C10	239.5	38.01	23	2.78E+06	18	26	58827.09
A0A2R8Y5G6|A0A2R8Y5G6_HUMAN	A0A2R8Y5G6	236.34433	32.32	20	9.85E+05	20	22	69047.84
A0A2R8Y645|A0A2R8Y645_HUMAN	A0A2R8Y645	236.34433	28.07	20	9.85E+05	20	22	79474.95
A0A2R8Y4A4|A0A2R8Y4A4_HUMAN	A0A2R8Y4A4	236.34433	30.36	20	9.85E+05	20	22	73243.41
A0A2U3TZJ9|A0A2U3TZJ9_HUMAN	A0A2U3TZJ9	236.34433	30.41	20	9.85E+05	20	22	73214.38
A0A2R8YFR4|A0A2R8YFR4_HUMAN	A0A2R8YFR4	236.34433	30.45	20	9.85E+05	20	22	73127.3
A0A0D9SF53|A0A0D9SF53_HUMAN	A0A0D9SF53	236.34433	27.42	20	9.85E+05	20	22	81477.03
A0A0D9SG12|A0A0D9SG12_HUMAN	A0A0D9SG12	236.34433	31.07	20	9.85E+05	20	22	71540.8
A0A0D9SFB3|A0A0D9SFB3_HUMAN	A0A0D9SFB3	236.34433	31.41	20	9.85E+05	20	22	70840.03
A0A2R8YFS5|A0A2R8YFS5_HUMAN	A0A2R8YFS5	236.34433	30.41	20	9.85E+05	20	22	73156.34
O00571|DDX3X_HUMAN	DDX3X	236.34433	30.36	20	9.85E+05	20	22	73243.41
A0A2R8YCW1|A0A2R8YCW1_HUMAN	A0A2R8YCW1	236.34433	31.07	20	9.85E+05	20	22	71693.75
A0A2R8YF78|A0A2R8YF78_HUMAN	A0A2R8YF78	236.34433	31.36	20	9.85E+05	20	22	70940.16
A0A1X7SBZ2|A0A1X7SBZ2_HUMAN	A0A1X7SBZ2	216.00574	18.11	14	2.01E+05	7	15	80254.41
Q92841|DDX17_HUMAN	DDX17	216.00574	18.11	14	2.01E+05	7	15	80272.44
A0A5H1ZRQ2|A0A5H1ZRQ2_HUMAN	A0A5H1ZRQ2	216.00574	18.06	14	2.01E+05	7	15	80439.63

Q13283|G3BP1_HUMAN	G3BP1	210.93777	37.98	13	4.72E+06	11	28	52164.24
P35527|K1C9_HUMAN	K1C9	209.76404	25.2	17	1.03E+06	16	17	62064.32
P61978|HNRPK_HUMAN	HNRPK	207.96867	33.69	14	6.23E+05	14	15	50976.24
P52272|HNRPM_HUMAN	HNRPM	206.59457	20.14	14	4.39E+05	14	14	77515.52
A0A087X0X3|A0A087X0X3_HUMAN	A0A087X0X3	206.59457	20.14	14	4.39E+05	14	14	77569.59
E9PKE3|E9PKE3_HUMAN	E9PKE3	204.92412	20.73	12	2.61E+05	6	12	68805.79
P11142|HSP7C_HUMAN	HSP7C	204.92412	20.12	12	2.61E+05	6	12	70898.09
O60506|HNRPQ_HUMAN	HNRPQ	202.73567	22.31	14	2.89E+05	8	16	69602.62
Q92804|RBP56_HUMAN	RBP56	198.96999	17.23	11	7.18E+05	8	14	61829.94
A0A6Q8PFJ0|A0A6Q8PFJ0_HUMAN	A0A6Q8PFJ0	196.9164	19.07	14	1.81E+05	14	14	80903.3
A0A6Q8PHQ9|A0A6Q8PHQ9_HUMAN	A0A6Q8PHQ9	196.9164	26.94	14	1.81E+05	14	14	58496.4
P02545|LMNA_HUMAN	LMNA	196.9164	20.93	14	1.81E+05	14	14	74139.48
O43390|HNRPR_HUMAN	HNRPR	189.90715	18.01	11	1.96E+05	5	11	70943.07
O00425|IF2B3_HUMAN	IF2B3	188.2633	17.62	10	8.78E+04	7	10	63705.03
B1AHC9|B1AHC9_HUMAN	B1AHC9	185.8196	16.46	9	2.26E+05	9	10	64283.53
P12956|XRCC6_HUMAN	XRCC6	185.8196	15.11	9	2.26E+05	9	10	69843.06
Q9Y6M1|IF2B2_HUMAN	IF2B2	184.2405	17.86	10	1.39E+05	7	10	66121.41
F8W930|F8W930_HUMAN	F8W930	184.2405	17.69	10	1.39E+05	7	10	66786.22
B0QY89|B0QY89_HUMAN	B0QY89	180.18402	13.34	9	1.17E+05	9	9	70901.82
Q9Y262|EIF3L_HUMAN	EIF3L	180.18402	14.36	9	1.17E+05	9	9	66726.98
Q96PK6|RBM14_HUMAN	RBM14	179.58833	11.51	8	1.57E+05	8	8	69491.64
P0DMV8|HS71A_HUMAN	HS71A	178.33302	13.42	8	6.30E+03	1	8	70052.23
P0DMV9|HS71B_HUMAN	HS71B	178.33302	13.42	8	6.30E+03	1	8	70052.23

A0A0G2JIW1|A0A0G2JIW1_HUMAN	A0A0G2JIW1	178.33302	13.4	8	6.30E+03	1	8	70109.28
Q07666|KHDR1_HUMAN	KHDR1	171.91734	15.35	9	1.07E+06	9	11	48227.33
Q9UN86|G3BP2_HUMAN	G3BP2	169.31244	17.43	7	3.54E+05	5	8	54121.13
Q9NUQ6|SPS2L_HUMAN	SPS2L	166.60323	12.19	7	1.31E+05	7	7	61729
H3BPE7|H3BPE7_HUMAN	H3BPE7	165.97533	11.95	6	2.65E+06	3	11	53496.91
P35637|FUS_HUMAN	FUS	165.97533	11.98	6	2.65E+06	3	11	53425.83
P13647|K2C5_HUMAN	K2C5	164.95134	7.8	7	3.67E+04	2	7	62378.34
P02533|K1C14_HUMAN	K1C14	153.76372	11.86	6	1.43E+04	2	6	51561.47
A0A1W2PPH7|A0A1W2PPH7_HUMAN	A0A1W2PPH7	148.5127	10.93	8	1.23E+05	8	9	61749.93
Q5RI18|Q5RI18_HUMAN	Q5RI18	148.5127	9.97	8	1.23E+05	8	9	67824.56
A0A1W2PP34|A0A1W2PP34_HUMAN	A0A1W2PP34	148.5127	10.99	8	1.23E+05	8	9	61052.43
Q00839|HNRPU_HUMAN	HNRPU	148.5127	7.27	8	1.23E+05	8	9	90584.43
A0A1W2PQL0|A0A1W2PQL0_HUMAN	A0A1W2PQL0	148.5127	11.39	8	1.23E+05	8	9	58820.53
A0A1W2PPS1|A0A1W2PPS1_HUMAN	A0A1W2PPS1	148.5127	7.46	8	1.23E+05	8	9	88318.07
A0A1W2PPL4|A0A1W2PPL4_HUMAN	A0A1W2PPL4	148.5127	10.97	8	1.23E+05	8	9	61563.77
A0A1X7SBS1|A0A1X7SBS1_HUMAN	A0A1X7SBS1	148.5127	8.06	8	1.23E+05	8	9	81794.84
Q6ZUT6|CCD9B_HUMAN	CCD9B	141.32599	8.43	5	6.17E+04	5	5	57324.85
P38646|GRP75_HUMAN	GRP75	139.55334	7.51	5	5.09E+04	4	5	73680.49
E9PLA9|E9PLA9_HUMAN	E9PLA9	136.28592	22.04	4	7.74E+04	4	4	20235.79
Q14444|CAPR1_HUMAN	CAPR1	136.28592	5.78	4	7.74E+04	4	4	78366.21
O15371|EIF3D_HUMAN	EIF3D	132.93745	5.84	4	5.81E+04	4	4	63972.77
B4DXZ6|B4DXZ6_HUMAN	B4DXZ6	124.95628	8.72	5	1.49E+05	5	5	68327.22
P51114|FXR1_HUMAN	FXR1	124.95628	8.53	5	1.49E+05	5	5	69720.78

E9PFF5|E9PFF5_HUMAN	E9PFF5	124.95628	10.82	5	1.49E+05	5	5	55105.66
E7EU85|E7EU85_HUMAN	E7EU85	124.95628	11.67	5	1.49E+05	5	5	50990.98
F5H5U2|F5H5U2_HUMAN	F5H5U2	110.65283	5.62	3	9.66E+03	3	3	65190.98
Q8NHQ9|DDX55_HUMAN	DDX55	110.65283	5.33	3	9.66E+03	3	3	68546.81
Q86YZ3|HORN_HUMAN	HORN	95.47392	0.81	2	8.80E+03	2	2	282389.9
A0A087X2G1|A0A087X2G1_HUMAN	A0A087X2G1	78.21218	4.55	3	6.73E+04	3	3	73975.36
F1T0B3|F1T0B3_HUMAN	F1T0B3	78.21218	4.55	3	6.73E+04	3	3	73915.34
Q92499|DDX1_HUMAN	DDX1	78.21218	4.05	3	6.73E+04	3	3	82432.14
P68032|ACTC_HUMAN	ACTC	77.42659	4.51	2	2.95E+04	2	2	42018.97
P63261|ACTG_HUMAN	ACTG	77.42659	4.53	2	2.95E+04	2	2	41792.84
P62736|ACTA_HUMAN	ACTA	77.42659	4.51	2	2.95E+04	2	2	42008.95
P68133|ACTS_HUMAN	ACTS	77.42659	4.51	2	2.95E+04	2	2	42051.03
A6NL76|A6NL76_HUMAN	A6NL76	77.42659	6.69	2	2.95E+04	2	2	28165.33
P63267|ACTH_HUMAN	ACTH	77.42659	4.52	2	2.95E+04	2	2	41876.87
P60709|ACTB_HUMAN	ACTB	77.42659	4.53	2	2.95E+04	2	2	41736.73
G3XAC6|G3XAC6_HUMAN	G3XAC6	70.8395	6.15	2	2.12E+04	2	2	48023.77
Q14498|RBM39_HUMAN	RBM39	70.8395	4.91	2	2.12E+04	2	2	59379.58
P35579|MYH9_HUMAN	MYH9	67.77671	0.71	1	1.00E+04	1	1	226532.2
P81605|DCD_HUMAN	DCD	67.18275	10	1	2.80E+04	1	1	11283.86
M0QYR1|M0QYR1_HUMAN	M0QYR1	66.0377	9.57	1	4.37E+03	1	1	11475.93
P08621|RU17_HUMAN	RU17	66.0377	2.06	1	4.37E+03	1	1	51556.68
Q96GA3|LTV1_HUMAN	LTV1	63.844944	2.53	1	5.27E+03	1	1	54854.93
Q14258|TRI25_HUMAN	TRI25	59.884705	1.9	1	1.05E+04	1	1	70973.4

A0A3B3IUA7|A0A3B3IUA7_HUMAN	A0A3B3IUA7	59.884705	2.63	1	1.05E+04	1	1	51073.52
D6RIY6|D6RIY6_HUMAN	D6RIY6	58.240757	2.6	1	6.50E+03	1	1	46977.73
Q06265|EXOS9_HUMAN	EXOS9	58.240757	2.51	1	6.50E+03	1	1	48949.02
H7C3E9|H7C3E9_HUMAN	H7C3E9	56.839233	2.53	1	1.46E+03	1	1	44165.85
P19338|NUCL_HUMAN	NUCL	47.52019	1.27	1	5.98E+03	1	1	76614.41
Q6UXG8|BTNL9_HUMAN	BTNL9	44.7168	1.31	1	5.17E+03	1	1	59716.01
A2AE48|A2AE48_HUMAN	A2AE48	44.39418	4.44	1	2.24E+03	1	1	28665.91
Q86W50|MET16_HUMAN	MET16	38.54346	1.42	1	3.32E+04	1	2	63621.03
P21589|5NTD_HUMAN	5NTD	36.342506	2.96	1	5.60E+03	1	1	63367.74
Q9H361|PABP3_HUMAN	PABP3	32.322304	0.95	1	5.79E+03	1	1	70030.92
A0A087WTT1|A0A087WTT1_HUMAN	A0A087WTT1	32.322304	1.15	1	5.79E+03	1	1	58536.02
E7ERJ7|E7ERJ7_HUMAN	E7ERJ7	32.322304	0.99	1	5.79E+03	1	1	67138.77
H0YAP2|H0YAP2_HUMAN	H0YAP2	32.322304	4.58	1	5.79E+03	1	1	15164.08
P11940|PABP1_HUMAN	PABP1	32.322304	0.94	1	5.79E+03	1	1	70670.84
H0YAR2|H0YAR2_HUMAN	H0YAR2	32.322304	2.11	1	5.79E+03	1	1	32258.47
E7EQV3|E7EQV3_HUMAN	E7EQV3	32.322304	1.02	1	5.79E+03	1	1	65748.16
H0YAW6|H0YAW6_HUMAN	H0YAW6	32.322304	3.21	1	5.79E+03	1	1	20816.62
O95429|BAG4_HUMAN	BAG4	31.53381	2.41	1	5.38E+03	1	1	49594.07
A6XGL3|A6XGL3_HUMAN	A6XGL3	30.368086	3.38	1	6.39E+04	1	1	25398.81
Q9BYE2|TMPSD_HUMAN	TMPSD	30.368086	1.37	1	6.39E+04	1	1	63167.15
P07477|TRY1_HUMAN	TRY1	30.368086	3.24	1	6.39E+04	1	1	26558.08
E7EQ64|E7EQ64_HUMAN	E7EQ64	30.368086	3.07	1	6.39E+04	1	1	28122.86
A0A6Q8PFP7|A0A6Q8PFP7_HUMAN	A0A6Q8PFP7	29.227974	9.28	1	4.70E+05	1	1	10778.47

P16402|H13_HUMAN	H13	24.137178	4.98	1	1.62E+04	1	1	22349.9
P16403|H12_HUMAN	H12	24.137178	5.16	1	1.62E+04	1	1	21364.75
P10412|H14_HUMAN	H14	24.137178	5.02	1	1.62E+04	1	1	21865.27
Q02539|H11_HUMAN	H11	24.137178	5.12	1	1.62E+04	1	1	21842.09
Q8N3V7|SYNPO_HUMAN	SYNPO	21.391857	1.18	1	1.62E+04	1	1	99463.41
O14744|ANM5_HUMAN	ANM5	18.836193	2.04	1	5.93E+03	1	1	72683.87
E9PDS3|E9PDS3_HUMAN	E9PDS3	18.126623	1.42	1	6.72E+03	1	1	69080.73
Q13797|ITA9_HUMAN	ITA9	18.126623	0.87	1	6.72E+03	1	1	114488.6
D6RHC4|D6RHC4_HUMAN	D6RHC4	15.925045	1.63	1	5.21E+03	1	1	37524.24
Q8IWZ3|ANKH1_HUMAN	ANKH1	15.925045	0.24	1	5.21E+03	1	1	269457.5
E9PDP5|E9PDP5_HUMAN	E9PDP5	15.925045	0.38	1	5.21E+03	1	1	166743.6
